# Epigenetic modification of gene expression in cancer cells by terahertz demethylation

**DOI:** 10.1038/s41598-023-31828-w

**Published:** 2023-03-26

**Authors:** Hwayeong Cheon, Junho K. Hur, Woochang Hwang, Hee-Jin Yang, Joo-Hiuk Son

**Affiliations:** 1 Biomedical Engineering Research Center, Asan Medical Center, 88, Olympic-ro 43-gil, Songpa-gu, Seoul, 05505 Republic of Korea; 2 Department of Genetics, College of Medicine, Graduate School of Biomedical Sciences and Engineering, Hanyang University, Wangsimni-ro, Seongdong-gu, Seoul, 04763 Republic of Korea; 3 Department of Pre-Medicine, College of Medicine, Hanyang Institute of Bioscience and Biotechnology, Hanyang University, Wangsimni-ro, Seongdong-gu, Seoul, 04763 Republic of Korea; 4 Department of Neurosurgery, Seoul National University Boramae Medical Center, 20 Boramae-ro 5-gil, Dognjak-gu, Seoul, 07061 Republic of Korea; 5 Department of Physics, University of Seoul, 163, Seoulsiripdae-ro, Dongdaemun-gu, Seoul, 02504 Republic of Korea

**Keywords:** Genetics, Terahertz optics

## Abstract

Terahertz (THz) radiation can affect the degree of DNA methylation, the spectral characteristics of which exist in the terahertz region. DNA methylation is an epigenetic modification in which a methyl (CH_3_) group is attached to cytosine, a nucleobase in human DNA. Appropriately controlled DNA methylation leads to proper regulation of gene expression. However, abnormal gene expression that departs from controlled genetic transcription through aberrant DNA methylation may occur in cancer or other diseases. In this study, we demonstrate the modification of gene expression in cells by THz demethylation using resonant THz radiation. Using an enzyme-linked immunosorbent assay, we observed changes in the degree of global DNA methylation in the SK-MEL-3 melanoma cell line under irradiation with 1.6-THz radiation with limited spectral bandwidth. Resonant THz radiation demethylated living melanoma cells by 19%, with no significant occurrence of apurinic/apyrimidinic sites, and the demethylation ratio was linearly proportional to the power of THz radiation. THz demethylation downregulates *FOS*, *JUN*, and *CXCL8* genes, which are involved in cancer and apoptosis pathways. Our results show that THz demethylation has the potential to be a gene expression modifier with promising applications in cancer treatment.

## Introduction

DNA methylation is an epigenetic mechanism in which a methyl group (CH_3_−) is attached to the fifth carbon of the cytosine ring in DNA, thereby regulating gene expression^1–3^. Methylated cytosine modulates gene expression by inhibiting transcription^4–7^. In normal cells, DNA methylation acts as a gene switch. Most cytosine-phosphate-guanine clusters (known as CpG islands) control gene expression by promoter methylation (activating expression) or demethylation (deactivating expression). When the regulatory mechanism of gene expression is disordered due to aberrant DNA methylation, gene-related diseases may occur (Suppl. Fig. 1). Inhibition of cancer suppressor genes by DNA methylation is known to be related to carcinogenesis^8–10^. Many studies have indicated an association between aberrant DNA methylation and alterations in gene expression in several cancer types, including melanoma, colon, lung, breast, ovary, bladder, liver, and cervical cancers^11–22^.

Aberrant DNA methylation contributes to abnormal genetic phenotypes similar to genetic mutations in cancer. Although alteration of genome sequences is generally irreversible, the demethylation process may have the potential to reverse DNA methylation (by placing the appropriate methyl groups at the proper cytosine). On May 19, 2004, Vidaza^TM^ (5-Azacytidine) became the first US FDA-approved methylation-inhibiting agent for the treatment of myelodysplastic syndrome (MDS)^23,24^. Decitabine^TM^ (2′-dexoy-5-azacytidine) has also been approved by the US and EU for treatment of MDS and acute myeloid leukemia (AML)^24,25^. Demethylation drugs inhibit all isoforms of DNMT and induce various types of tumor suppressors. These dilution processes of DNA methylation depend on genomic heredity and are referred to as passive demethylation (Suppl. Fig. 4).

In contrast, active DNA demethylation refers to an enzymatic process that removes methyl groups directly from cytosine. The mechanism of active DNA methylation is not yet fully understood; however, almost all DNA regions are demethylated by the activation of enzymes during reprogramming. In particular, the ten–eleven translocation (TET) family of methylated cytosine dioxygenases is used independently for active demethylation^26–28^. Consequently, the activity of TET enzymes exchanges 5-mC for nonmethylated cytosine directly, completing demethylation (Suppl. Fig. 5). TET inactivation can result in hypermethylation of the tumor suppressor genes, leading to carcinogenesis. Therefore, several researchers are exploring the development of novel drugs to regulate the TET enzyme; however, no TET protein inhibitors have been clinically tested for cancer treatment.

Terahertz (THz) radiation has relatively low energy (i.e., insufficient to cause ionization), but it is highly sensitive to the chemical binding energy of various biomolecules^29–33^. DNA methylation is a biochemical modification of DNA that does not involve a change in sequence, and the characteristic energy of methylated DNA is observed in the THz region. Thus, THz radiation with proper intensity and frequency can modify the bonding conditions of methylated DNA^34^. In terms of directly reducing DNA methylation, similar to active demethylation, THz demethylation may be proposed as an interventional method for cancer treatment. There have been numerous studies on aberrant DNA methylation in melanoma^11,35–37^. This is the most promising type of cancer for research on biomedical applications of THz radiation for treatment because of the easy accessibility of malignant lesions. Manipulation of DNA methylation to correct abnormalities in malignant lesions may be an effective approach to molecular cancer therapy. We showed evidence of a specific spectral resonance of methylated DNA in the THz region by comparing artificially methylated DNA and nonmethylated DNA. In addition, the quantified results of the degree of global methylation in several solid cancers correlated with the intensity of the spectral resonance^34^. Spectral resonance was also observed in DNA from several blood cancers, and high-power THz radiation, which has a specific energy corresponding to the resonant frequency, reduced the degree of methylation in DNA isolated from blood cancers^38^. We termed the reduction of the methylation degree using resonant THz radiation “THz demethylation” and reported the effective conditions of irradiation power and exposure time for cancerous cells in our previous study^39^. THz demethylation achieves active demethylation using resonant high-power THz radiation restricted to a narrow band spectrum surrounding the molecular resonance in the THz frequency region (Suppl. Fig. 2).

In this study, we demonstrate the modification of gene expression in living melanoma cells by THz demethylation. THz radiation reduces the degree of methylation, which is accompanied by changes in gene expression. Genes showing changes in expression in response to THz radiation are associated with cancer pathways. According to this study, THz radiation can affect biological activity in living cells, although it is non-ionizing radiation that does not damage DNA sequences. These findings imply that THz demethylation, a technique that uses electromagnetism to modulate epigenetics, may have potential applications in molecular therapy for cancer.

## Results

### Power-dependence of THz demethylation in living cells and apurinic/apyrimidinic (AP) sites

**Figure 1:**
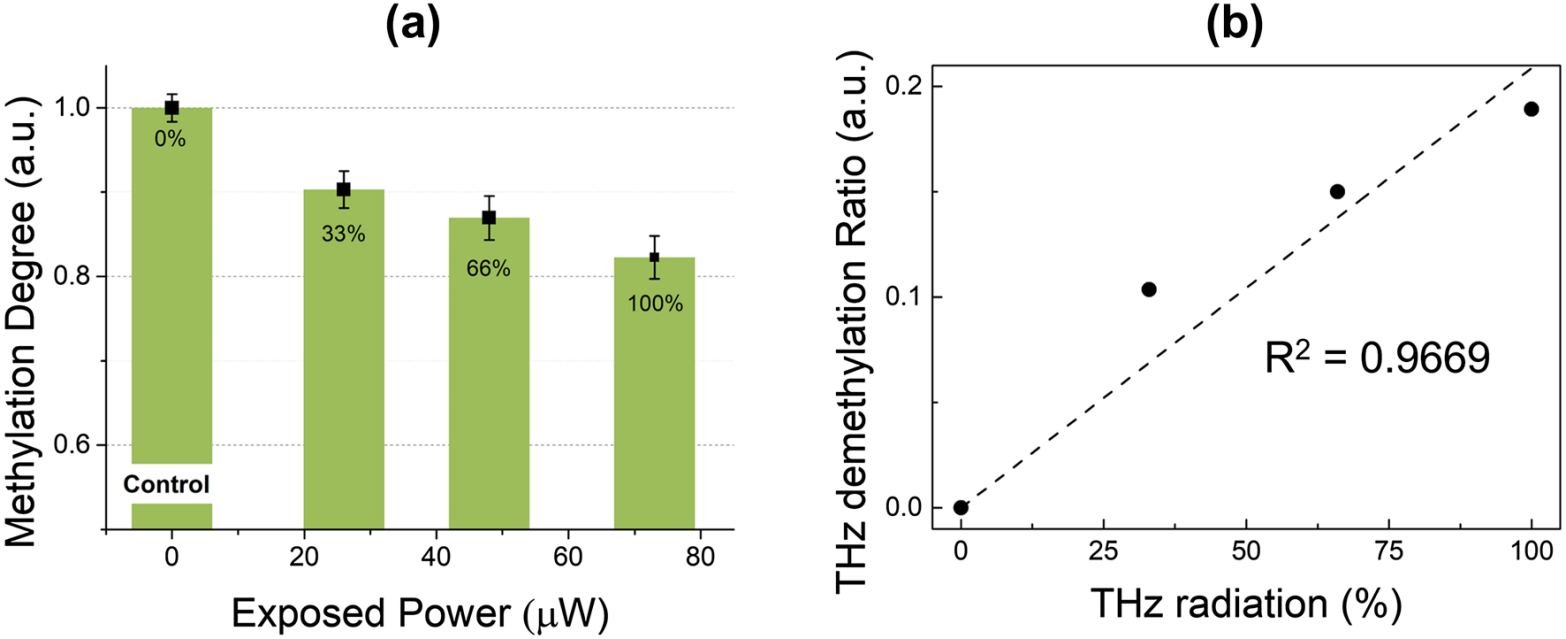
Power dependency of THz demethylation in living SK-MEL-3 melanoma cells. (**a**) The methylation degree decreased with the increasing power of resonant THz radiation. (**b**) The relationship between the THz demethylation ratio and the power of resonant THz radiation. The relationship demonstrated a linear dependency (R^2^ = 0.9669).

To assess the correlation between the power of THz radiation and the degree of reduction in global DNA methylation in living cells, we observed the power dependency of THz demethylation in SK-MEL-3 living cell pellets at a controlled exposure time of 30 min. The number of cells in the cell pellet and the focal position of the THz radiation were controlled to obtain a constant absorption cross-section in the samples. As THz radiation passes through the flat bottom of a polypropylene centrifuge tube, hardly any radiation is attenuated because polypropylene has a low absorption coefficient (0.58–0.6 cm^−1^ at 1 THz)^40^. The methylation degree was measured in genomic DNA extracted from every four pairs of samples, which served as controls. Living cells were exposed to resonant THz radiation. The maximum power of 1.6-THz irradiation (73 μW) reduced the normalized degree of global methylation by approximately 19% compared to the control (non-irradiated) cells, as shown in Fig. 1. The degree of demethylation increased with the THz irradiation power. This result indicates that THz radiation can affect genomic DNA in living cells. It also indicates that THz demethylation has a power dependency, which is similar to the previous result of power dependency in isolated genomic DNA^38^. The most effective exposure time for THz demethylation in living cells is 30 min. We speculated that THz demethylation would require a longer exposure time than multiple photon dissociation by infrared waves. This is because THz waves have much lower energy than infrared waves (1.6 THz = 6.62 meV), and this exposure time may be required for the accumulation of energy above the dissociative level. The degree of methylation was assessed by averaging six optical density (OD) values from enzyme-linked immunosorbent assay (ELISA) quantification, in multiple experiments (n = 3), in a consistent environment.

**Figure 2:**
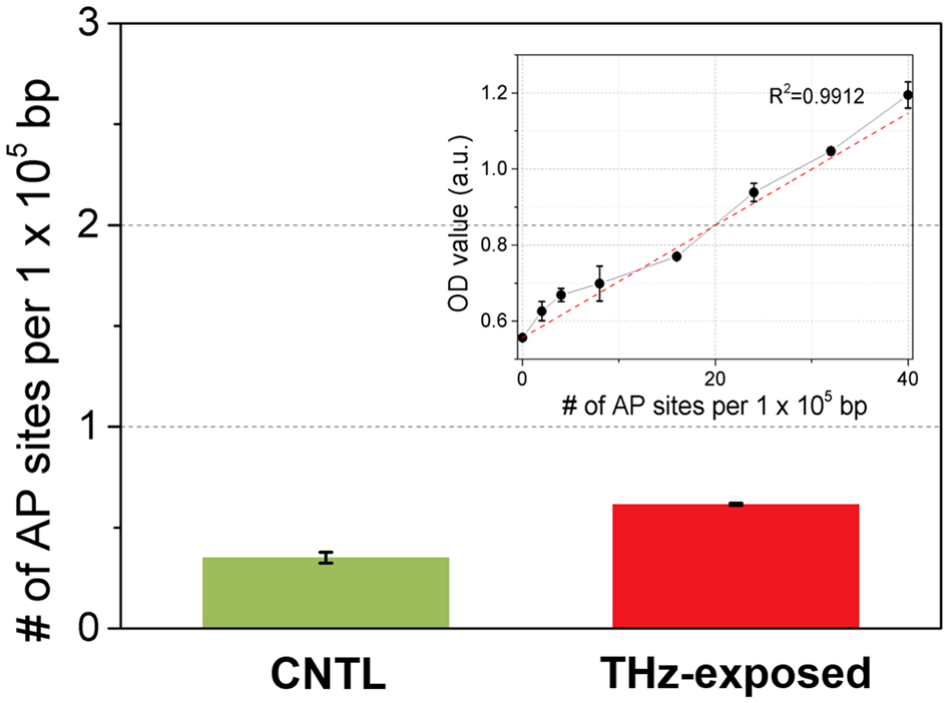
The number of AP sites in control (CTRL) living cell pellets without THz radiation and THz-exposed living cell pellets (THz-exposed) for SK-MEL-3. THz demethylation induced less than 1 AP site per 10^5^ bps, which was determined using a standard curve (inset graph).

The existence of apurinic/apyrimidinic (AP) sites due to DNA damage is an important issue for evaluating the effect of THz demethylation. High-power THz radiation used in THz demethylation may affect DNA structure directly, resulting in inaccurate detection of demethylated cytosine in the ELISA quantification process. Quantification of the AP sites was performed to confirm that no DNA damage occurred in response to THz radiation. According to Fig. 2, AP sites (DNA damage) had no effect on the degree of DNA methylation in THz-exposed cells despite the sample having been irradiated by the maximum power of THz radiation. Figure 2 shows that THz radiation induced a few AP sites (less than one AP site/10^5^ bp) in the THz-exposed cells (THz_expd). The number of AP sites per 10^5^ nucleotides was obtained by averaging six OD values from triplicate measurements. The number of AP sites was determined by referring to a standard curve established before quantification.

### Changes in DNA methylation degree and gene expression in living melanoma cells after THz demethylation

**Figure 3:**
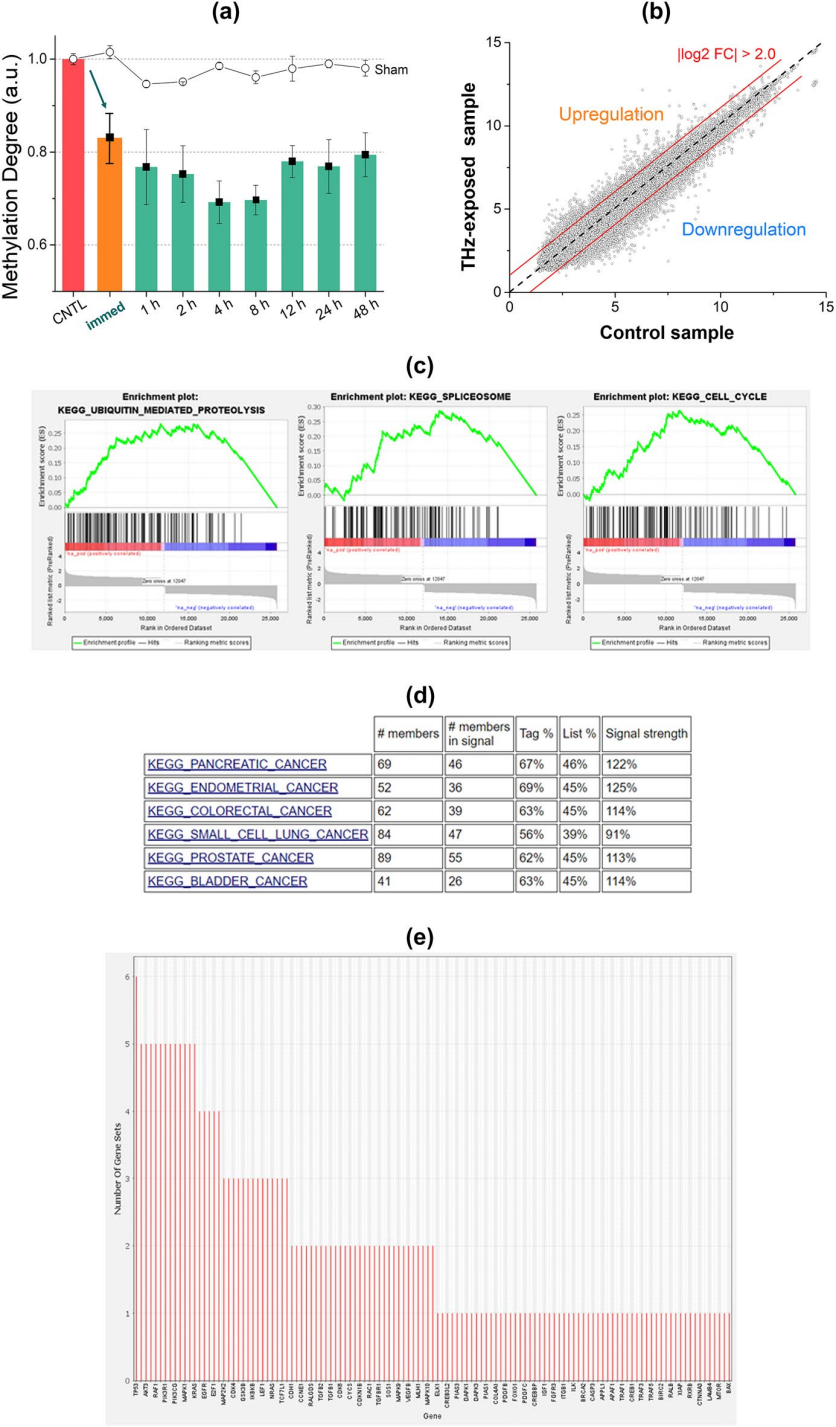
(**a**) Changes in the degree of DNA methylation after THz demethylation over time in SK-MEL-3 melanoma cells. The sham group contained non-irradiated cells that otherwise underwent the same treatment process. (**b**) An abundance plot showing the distinction of genes between THz-exposed and control samples in the immediate (indicated as “immed”) condition. A fold change of two was used as a cut-off point for identifying up- or down-regulated genes. (**c**) Gene set enrichment analysis (GSEA) of pathways with the highest enrichment scores: ubiquitin-mediated proteolysis, spliceosome, and cell cycle. (**d**) Leading-edge analysis of GSEA for genes with reduced expression levels by THz demethylation. Six cancer pathways contributed the largest enrichment signals. (**e**) Genes in the six cancer pathways. Some genes are commonly included in multiple pathways, including cancer pathways.

Considering DNA methylation is a pivotal mechanism of epigenetic modification that is heavily involved in the regulation of gene expression, we anticipated that THz demethylation could modify gene expression in living melanoma cells. Before observing changes in gene expression, we investigated the alterations in the global methylation degree over time following THz demethylation. In this experiment, the cells were exposed to a maximum power of THz radiation for 30 min and collected at specific intervals from 0 to 48 h. The 0 h means that the sample was collected and measured immediately after THz irradiation, and we called it “immed” in this study. We cultured the cells after THz irradiation and collected the same number of cells from each dish at different times to measure the degree of methylation. Since the population doubling time of SK-MEL-3 cells is known to be 40–50 h^41^, the degree of methylation was observed until the doubling time. THz demethylation in SK-MEL-3 cells occurred immediately after irradiation, and the degree of methylation did not recover to that of the control or sham groups, as shown in Fig. 3a. The sham group comprised samples that underwent the same process as the experimental sample, except for THz radiation.

We examined whether THz demethylation at 0 h (the "immed" sample) influenced the expression profile of early response genes, as well as the proliferation and apoptosis pathways of cancer cells. Therefore, we analyzed the RNA expression levels of 44,629 genes and observed that the expression levels of certain genes were up- or down-regulated by more than twofold following THz demethylation (Fig. 3b). Next, we conducted gene set enrichment analysis (GSEA) using KEGG pathways to investigate whether specific pathways were associated with changes in gene expression levels. GSEA of genes with reduced expression levels due to THz demethylation showed that 98 pathways, including 13 cancer pathways, were potentially enriched (Suppl. Table 1). The pathways with the highest enrichment scores included ubiquitin-mediated proteolysis, splicing, and the cell cycle, suggesting that THz demethylation may affect early response genes related to cell proliferation (Fig. 3c). Notably, six cancer pathways were included in the top 38 enriched pathways with a false discovery rate q-value of less than 0.25 (Fig. 3d). We visualized a leading-edge subset to identify genes that contributed the most to the enrichment signal of the GSEA and found that six cancer pathways encompassed 62 genes (Fig. 3e).

### THz demethylation effect on late-response genes in melanoma cells: transcription factor genes (FOS/JUN) and an inflammation-related gene (CXCL8)

**Figure 4:**
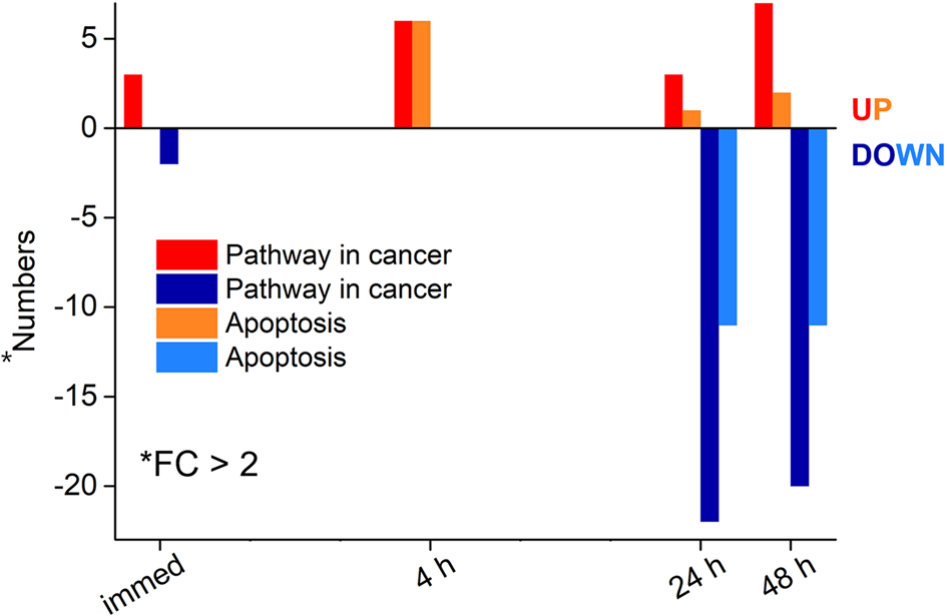
The number of up- and down-regulated genes in cancer and apoptosis pathways, as obtained from KEGG pathway analysis. The red and orange bars represent up-regulation of pathway in cancer and apoptosis, respectively, while the blue and navy-blue bars show down-regulation of pathway in cancer and apoptosis, respectively.

We sought to assess the effect of the 30 min process of THz demethylation on living cells over 48 h. As a result, we investigated the up-and-down-regulation of genes in the cancer and apoptosis pathway based on mRNA obtained at 0 h (“immed” sample), 4 h, 24 h, and 48 h. Up- and down-regulation refers to an increase or decrease in the activity of specific genes in response to an external stimulus (in this case, THz radiation), and gene modification may induce a biological response in cells. The cutoff of a twofold change (FC), which refers to the ratio between the control and THz samples (2-FC = 2 times), was used to select significant differentially expressed genes (DEGs) that provide meaningful data, as shown in Fig. 3b. Figure 4 displays the number of upregulated and downregulated genes in the cancer and apoptosis pathways from the KEGG pathway database. Although many genes exhibited altered expression patterns, the downregulation of gene expression in both the cancer and apoptosis pathways after 24 h was conspicuous. To show the changes in gene expression in detail, we analyzed some of the identified genes at each time point. In the cancer and apoptosis pathways, the *JUN* and *FOS* gene families and the *CXCL8* gene were downregulated significantly (more than 4-FC) after 24 h, in both the cancer and apoptosis pathways, despite changes in the expression of several genes (Suppl. Fig. 3).

**Figure 5:**
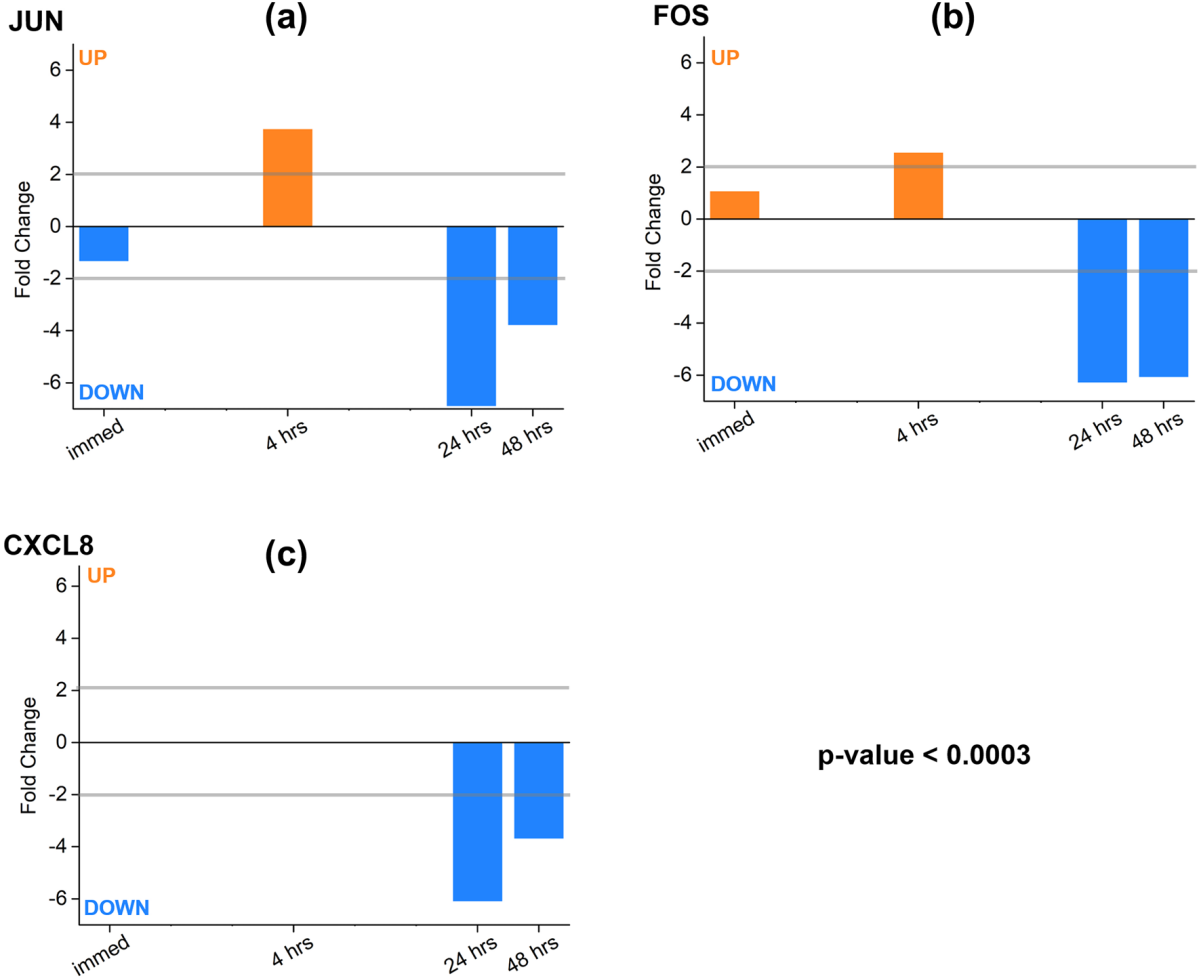
Modification of expression in the (**a**) *JUN*, (**b**) *FOS*, and (**c**) *CXCL8* genes after THz demethylation in SK-MEL-3 melanoma cell pellets. After 24 h of THz exposure, downregulation with a fold-change over four occurred in these genes (*p*-value < 0.0003).

We focused on the expression levels of *FOS*, *JUN*, and *CXCL8*, which were significantly altered by THz demethylation. *FOS* and *JUN* genes encode proteins that dimerize to form activator protein (AP)-1 transcription factors that regulate differentiation, proliferation, and apoptosis^42–44^. *CXCL8* is a major transcription and inflammation factor in the progression of melanoma^45–47^. We analyzed the modification of the *FOS*, *JUN*, and *CXCL8* genes after THz demethylation. *FOS* and *JUN* showed similar findings after 24 h, as shown in Fig. 5. THz exposure upregulated the expression of these genes at 4 h; however, the expression decreased significantly over 4-FC after 24 h (Suppl. Fig. 3). The results suggested that THz demethylation temporarily stimulated transcription factors and gradually re-adjusted the abnormal overexpression of the affected genes.

## Discussion

DNA methylation and demethylation have significant potential in cancer diagnostics and therapeutics owing to the role of DNA methylation in regulating gene expression. It is plausible that the manipulation of DNA methylation has a considerable impact on cancer, the initiation and progression of which involve alterations in DNA methylation. However, many aspects of the mechanisms of epigenetic modifications are not fully understood, and their potential will not come to fruition until after these gaps in knowledge are clarified.

We have discussed the relationship between THz radiation and DNA methylation from DNA molecules to cells in several publications^20,21,39,48,49^. In this study, we demonstrated the modification of the methylation degree and gene expression by THz demethylation in living SK-MEL-3 melanoma cells. The effectiveness of THz demethylation in living cells increased with the irradiation power, similar to the results observed for isolated DNA and DNA in cell structures. The global methylation degree was reduced by approximately 20% in the maximal THz radiation of our system (73 µW for 30 min), whereas there was less than one AP site per 10^5^ bps. Particularly, the difference of mRNA levels of DNMT1 between control and THz exposed samples was not significant in our microarray data, which was only 1.091-fold difference. This indicates that THz demethylation occurs independently of DNMT enzyme activity. THz demethylation affected the degree of methylation in cells for at least 48 h and modified the expression of genes in cancer and apoptosis pathways. Significant downregulation of the *FOS, JUN*, and *CXCL8* genes was observed in response to THz demethylation, particularly after 24 h.

JUN and FOS proteins contain activator protein 1 (AP-1) transcription factors that are homo- and heterodimers^50^. AP-1 transcription factors are essential components of transforming growth factor-β (TGF-β)-SMAD signaling during pro-oncogenic progression (i.e., cancer cell growth)^51^. Therefore, the *JUN* and *FOS* gene families contribute significantly to cancer transformation and cell growth. When *FOS* and *JUN* are overexpressed, AP-1 transcription increases, leading to cell overgrowth (abnormal cell growth)^52–54^. Additionally, *CXCL8* is crucial in the progression of human malignant melanoma^55,56^. Considering that cell overgrowth is essential to the development of cancer cells, suppressing these genes could prevent cancer growth and progression. We hypothesized that THz demethylation induced transcription factor gene upregulation by *FOS* and *JUN* at 4 h, and the transcription factor genes were reprogrammed at 24 h, as observed by their significant downregulation. The mechanism of THz demethylation remains under investigation, both physically and biologically. As an alternative, it might be developed into a unique non-contact technique for modifying gene expression in living cells by achieving active demethylation. Furthermore, active demethylation removes methyl groups from DNA independently of replication (Suppl. Fig. 6).

There were some limitations in this research. First of all, there is a potential that THz radiation might affect other related life activity, such as TET activity or histone modification, besides DNA methylation although we examined the microarray data that terahertz irradiation did not affect DNMT activity. In previous studies, THz demethylation occurred even in conditions excluding cell activity, such as isolated DNA or DNA in frozen cell structures^38,39^. We suspect that THz demethylation may have affected gene expression, because DNA methylation is an epigenetic gene regulator; however, further investigation is required. In addition, the underlying mechanism of THz demethylation in living cells has not yet been clarified. In repeated experiments under various conditions, we expect that there is a resonance frequency associated with methylated DNA in the THz region. However, further research is needed to determine how methyl molecules are removed from DNA within cellular structures.

Although additional studies are required for clinical application, the THz demethylation technique might be an effective alternative to demethylation methods for epigenetic cancer therapy. In clinical practice, some drugs for passive demethylation have been approved, and these drugs reprogram the aberrant regulation of gene expression for an anti-cancer effect at appropriate doses. However, the mechanism of demethylation remains unclear, and there are concerns regarding its side effects. The most significant problem is the possibility of increasing mutagenic risks and genomic instability. These drugs can cause nausea, low red blood cell count (anemia), and an increased risk of bleeding, vomiting, fever, and low white blood cell count^57,58^. Currently, active demethylation is not available as a technique or as a therapeutic agent in clinical practice. Considering the history of active demethylation is relatively shorter than that of passive demethylation, further research into active methylation mechanisms is needed. Although THz demethylation is not involved in enzymatic activity unlike the existing method for active demethylation using the enzymes, we think that it may be used to study the effect and mechanism of active demethylation in terms of directly removing methylation from the DNA molecules. In addition, the biomedical engineering techniques for epigenetic research may contribute to develop new techniques of active demethylation technologies for cancer treatment in clinical practice.

## Methods and materials

There were no statistical methods used to determine the sample size, and none of the samples were excluded from the analysis. All experiments were performed in accordance with the relevant guidelines and regulations of the University of Seoul and the Seoul Metropolitan Government Boramae Medical Center.

### Experimental system setup for THz demethylation

**Figure 6:**
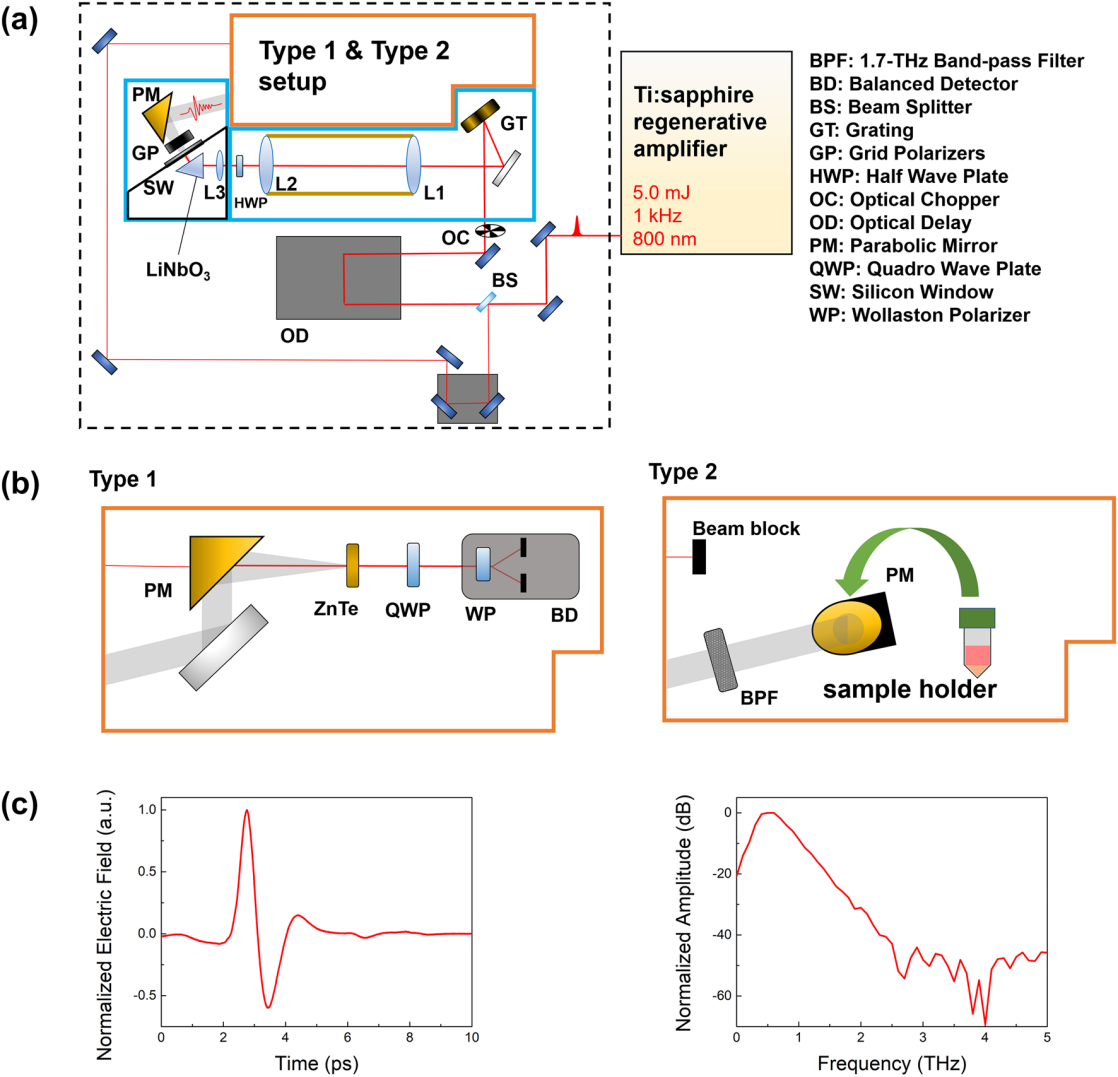
(**a**) Schematic of the THz generation and detection system for demethylation using resonant THz radiation. (**b**) Two types of THz setups for this study. High-power THz radiation was generated using a regenerative amplifier and a LiNbO_3_ crystal and detected with a ZnTe crystal in a type-1 setup. In the type-2 setup, for THz demethylation, the THz bandpass filter limited the bandwidth around the resonance frequency of the methyl-DNA bonds. (**c**) The waveform and spectrum of THz radiation which was measured by the type-1 setup.

A regenerative amplifier (Solstice Ace; Spectra-Physics, Irvine, CA) system, which generates a laser beam of 5.0 mJ per pulse with a 1-kHz repetition rate at an 800-nm wavelength, was used for the experimental laser system. The amplifier was injected with a seed beam from a Ti:sapphire oscillator (Mai-Tai; Spectra-Physics, Milpitas, CA), which is a 35-femtosecond pulsed laser (84 MHz repetition rate) with an average power of 750 mW at an 800-nm wavelength. The amplified beam was pumped by a 527-nm pump laser (Ascend; Spectra-Physics, Milpitas, CA) with a pump power of 25 W. The beam was separated by a polarizing beam splitter and turned towards the THz-generating crystal (99% of the total power) and the detecting part (1% of the total power) separately. The phase front of the THz-generating beam was tilted by a grating with a groove number of 2500 mm^−1^ for phase matching with the THz radiation. High-power THz radiation was generated by a MgO-LiNbO_3_ crystal excited by a tilted-phase-front beam. In addition, the crystal was tilted 62° to the beam front to achieve phase matching between the exciting laser beam and THz radiation. The power of the high-power THz pulse was approximately 14.7 mW/cm^2^, which was measured using a THz pyroelectric detector (T-Rad; Gentec-EO, Quebec City, Canada). The crystal area was covered by black-anodized coated metal plates, and the THz radiation could pass through a silicon window to block the residual infrared laser beam, thereby preventing accurate measurements. The THz radiation was passed through a pair of grid polarizers, which controlled the radiation power. Furthermore, a cross-shaped THz bandpass filter controlled the spectrum of THz radiation at the 1.6-THz center frequency with a 0.57-THz FWHM (Tydex, St. Petersburg, Russia) and affected the resonant molecular interactions with a specific energy. The resonant THz radiation was collected using a parabolic mirror. The THz radiation profile was measured using a type-1 setup, which included a ZnTe crystal, quarter-wave plate, Wollaston polarizer, and balanced detector (Fig. 6a,b). A type-2 setup was used to irradiate the cell sample with high-power THz radiation (Fig. 6b). The time-domain waveform and spectrum of THz radiation are shown in Fig. 6c.

**Figure 7:**
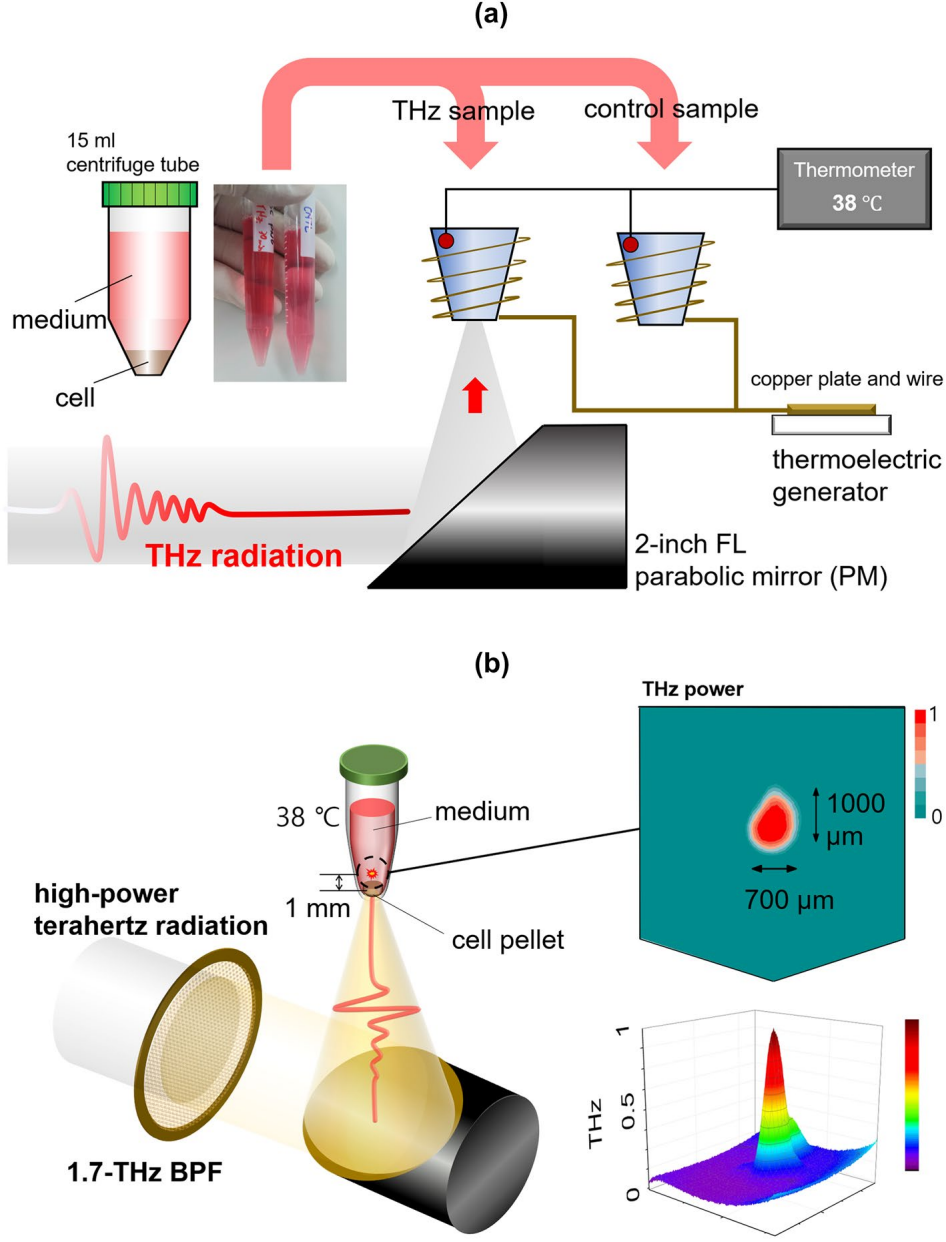
Schematic of the sample holder for THz demethylation. (**a**) The sample holder maintains a constant temperature at 38℃ with temperature sensors and a thermoelectric generator. (**b**) Melanoma cells were collected densely into living cell pellet samples by centrifugation. The bottoms of the tubes were irradiated with THz radiation to reduce THz attenuation by water molecules. The beam profile of THz radiation was measured using a pyroelectric detector and uncooled bolometer camera for THz waves.

THz radiation was focused vertically upward 1 mm above the bottom of 15-mL centrifuge tubes containing cells. Most cells (cell pellet) were located at the bottom of the tube, and THz radiation covered the whole area of the cell pellet. The temperature of the sample holder was controlled by a customized thermoelectric generator, and the samples were maintained at a temperature of 38℃ for living cells, as shown in Fig. 7a. In the experiment, we prepared two samples. One was THz-exposed, and the other was used as a sham sample to compare the degree of DNA methylation and gene expression. The sham sample was treated with the same methods, in the same environment, and at the same time with the exception of THz radiation. Although the THz radiation was attenuated heavily by water molecules, it irradiated the bottom of the tubes relatively evenly. We optimized the THz radiation in real time and measured the beam profile using a THz pyroelectric detector and an uncooled THz camera (IRV-T0831; NEC, Tokyo, Japan), as shown in Fig. 7b.

### Preparation of the SK-MEL-3 melanoma cell pellets

**Figure 8:**
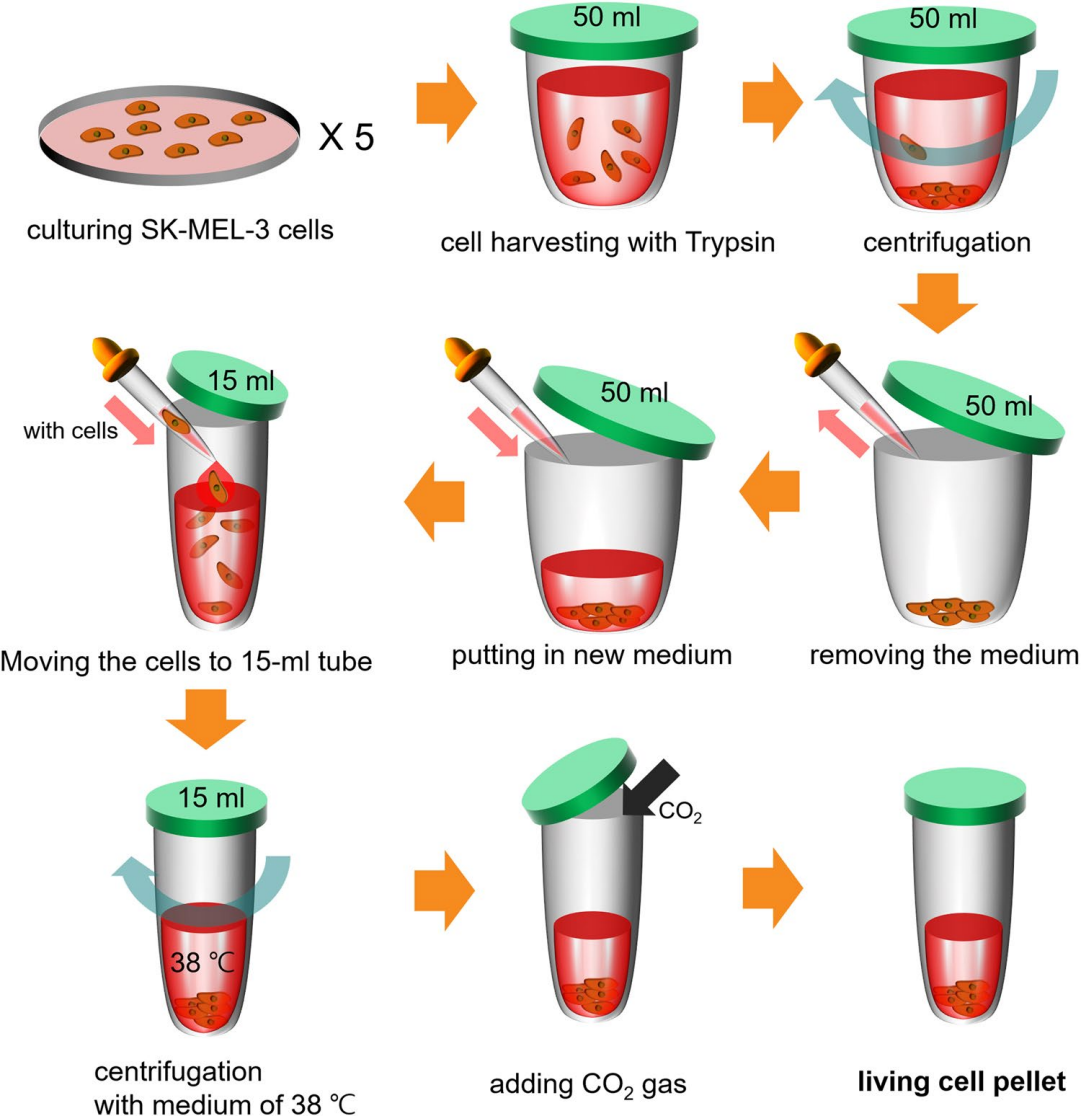
The production process of SK-MEL-3 melanoma living cell pellets. The cell pellets are primarily located at the bottom of tubes to ensure effective irradiation with THz radiation because of the absorption of THz radiation by water molecules.

The SK-MEL 3 melanoma cell line, established from a lymph node of a Caucasian woman, was obtained from the American Type Culture Collection (ATCC, Manassas, VA, USA). The cells were cultured in several 100-mm diameter cell culture dishes and plated at a density of 3 × 10^5^ cells/dish. To harvest the melanoma cells, we used trypsin (TrypLE Express, Gibco, Waltham, MA) to detach them from the dish surface, and the cells were collected in a 50-mL centrifuge tube. The cell culture medium was changed after centrifugation for 5 min at 1500 rpm, and the cells were transferred to a smaller tube (15 mL). The supernatant was discarded after centrifugation, the medium was heated to 38 °C, and a living cell pellet for the experiment was prepared. Each cell pellet was obtained from five dishes, and the total number of cells was 15 × 10^5^ cells in each tube (Fig. 8).

### Extraction of DNA samples for gene analysis

Purified DNA samples were used to measure the degree of methylation and the AP sites. Genomic DNA was extracted from SK-MEL-3 cells using the FavorPrep Blood/Cultured Cell Genomic DNA Extraction Mini Kit (FAVORGEN, Ping-Tung, Taiwan) according to the manufacturer’s protocol. Genomic DNA was dissolved in distilled water and purified to remove any residual material. The concentration of the DNA samples was measured using a NanoDrop ND-1000 UV–Vis spectrophotometer (Thermo Fisher Scientific Inc., Wilmington, DE), and the concentration of the DNA samples was 500 ± 8 µg/mL.

### ELISA quantification for global DNA methylation

In this study, we focused on the modification of global DNA methylation at the whole-DNA level and its impact on gene expression. The ELISA method is commonly used to measure the degree of methylation of whole genomic DNA. We assessed the degree of global DNA methylation using the Methylamp Global DNA Methylation Quantification Ultra Kit (Epigentek Inc., Farmingdale, NY, USA), which has the most references^59–62^. Previous researchers reported that this ELISA method is sensitive to global methylation in DNA above 100 bp length, and the light-absorbed antibody for ELISA detects as low as 0.2 ng of methylated parts in 50 ng of input genomic DNA. The OD was measured using an NS-100 Nano Scan Microplate Reader (Hercuvan Lab Systems, Cambridge, United Kingdom), and OD values were obtained through comparisons with the positive and negative controls according to the manufacturer’s protocol. The degree of DNA methylation was calculated from the normalization of the OD values compared to the value of the control sample.

### Assessment of DNA damage by examining AP sites

DNA damage induces AP sites that have missing bases (purine or pyrimidine bases). AP sites in living cells can cause cell death or severe mutations, and the number of AP sites can serve as an indicator of DNA damage caused by high-power THz radiation. To quantify AP sites in the genomic DNA of samples, we used the OxiSelect Oxidative DNA Damage Quantitation Kit, AP Sites (Cell Biolabs Inc., San Diego, CA, USA)^63,64^. The number of AP sites was determined by comparison with the standard curve fit according to the manufacturer’s protocol. The aldehyde reactive probe (ARP) in the kit was attached to an AP site, and the ARP was tagged with biotin/streptavidin-enzyme, which allows detection of the AP sites by OD values using the ELISA reader.

### Investigation of up-/down-regulation induced by THz demethylation

RNA was extracted from three biological replicates for differential gene expression analyses using the Affymetrix GeneChip Human 2.0ST microarray (Affymetrix, Santa Clara, CA). The Affymetrix GeneChip array was performed according to the manufacturer's protocol. RNA (1000 ng) labeled with the FlashTag Biotin RNA Labeling Kit (Genisphere, Hatfield, PA) was quantified, fractionated, and hybridized to the microarray according to standard procedures. RNA array hybridization was performed with agitation at 60 rotations per minute for 16 h at 48 °C on an Affymetrix GeneChip Hybridization Oven 645. The chips were stained using the GeneChip Fluidics Station 450 (Affymetrix, Santa Clara, CA, USA). The chips were then scanned with an Affymetrix GCS 3000 scanner (Affymetrix, Santa Clara, CA, USA) to measure gene expression.

The fold change (FC) in expression was measured using normalized expression levels for each gene. The FCs of genes with higher expression levels in the control sample were computed by dividing the expression levels in the control sample by those in the THz sample. The FCs of genes with higher expression levels in the THz sample were calculated as negative values of the ratio of THz sample expression levels to the expression levels in the control sample. Genes with an absolute fold change greater than two were identified as DEGs. KEGG pathway and gene ontology enrichment analyses were performed on the DEGs using GProfiler^65^. Gene set enrichment analysis (GSEA)^66^ was performed using all genes ranked by the FCs.

### Ethical standards

All procedures involving human cell lines were performed in accordance with the ethical approval of the institutional boards at the Seoul National University Boramae Medical Center.

## Supplementary Information

Supplementary Information.

## Data Availability

All data generated or analysed during this study are included in this published article [and its supplementary information files].
