# Supplementary material for: Epigenetic modification of gene expression in cancer cells by terahertz demethylation
Source: Sci Rep. 2023 Mar 26;13:4930. doi: 10.1038/s41598-023-31828-w (PMC10040409; doi:10.1038/s41598-023-31828-w)
Supplement: Supplementary file 1 — Supplementary Information. [file 41598_2023_31828_MOESM1_ESM.docx]

**Epigenetic modification of gene expression in cancer cells by terahertz demethylation**

Supplementary material

**DNA methylation in cancer**

The most common epigenetic process to regulate gene expression is DNA methylation in human DNA. DNA methylation is related to the conjunction of the methyl (CH_3_–) group on the 5^th^ carbon of cytosine (5-methylcytosine, 5-mC) by DNA methyltransferases (DNMTs) such as DNMT1, DNMT3a, and DNMT3b. The methyl groups in DNA play a role in a switch of gene expression. The genes become silent in the hypermethylated promoter, whereas hypomethylation activates the gene expression. The role of gene regulation is essential to maintain genetic stability and suppress the gene expression of certain cell–types in our body ^1–3^. Aberrant DNA methylation affects the cancer suppressor genes, and it may lead to changes in transcriptional pathways and cancer development. Therefore, DNA methylation is considered to be a carcinogenic biomarker and trigger for carcinogenesis. In recent research, the relationship between aberrant DNA methylation and gene alterations has been reported in several types of cancer including melanoma colon, lung, breast, ovarian, bladder, liver, and cervical cancer ^4–7^. In carcinogenesis, two types of epigenetic modifications are usually observed; hypermethylation of CpG islands and hypomethylation in the genome-wide region ^8^. Hypomethylation in the repeated DNA sequence of whole genomes induces genomic instability and increases the cancerous progress from non–metastatic to metastatic pathway ^9-11^. On the other hand, hypermethylation is associated with the gene silencing of tumor suppressors or transcription genes. Many researchers reported that multiple hypermethylated tumor suppressors were found in the cancer genome ^8,12,13^ (Suppl. Fig. 1). These epigenetic profiles in cancer DNA may be a target to achieve molecular cancer therapy.


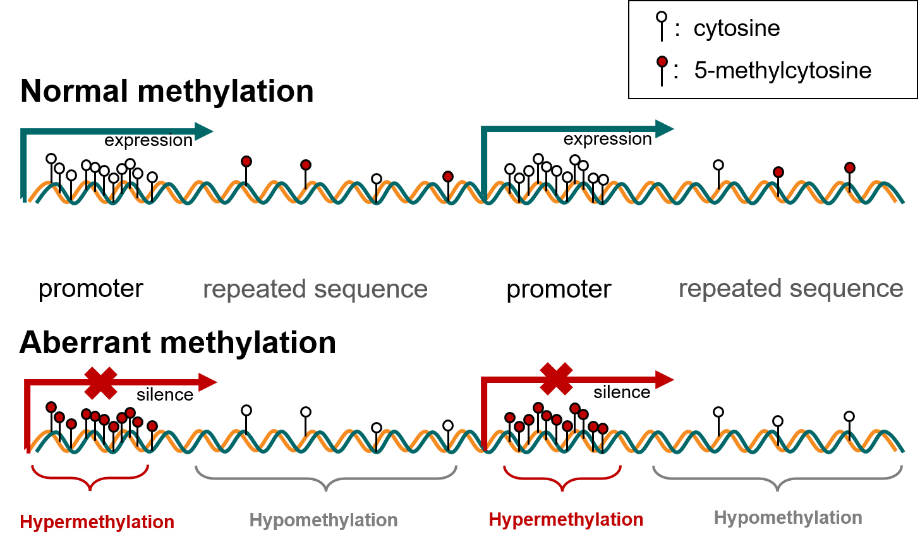


**Supplementary Figure 1** The pattern of hypermethylation and hypomethylation in aberrant DNA. The alternation of methylation distribution may lead to cause diseases like cancer. Especially, hypermethylation of tumor suppressor and genome-wide hypomethylation is significant hallmark features in carcinogenesis. Hypermethylation of tumor suppressors is associated with the generation of unleashed cell growth and hypomethylation induces genomic instability which may cause genomic mutation.

**THz demethylation**

Terahertz (THz) radiation is located between microwave and infrared radiations in the electromagnetic spectrum. Because the frequency band ranges from 0.1 to 10 THz (3.3 cm^-1^ – 330 cm^-1^ in wavenumber or 0.4 – 40 meV in energy), it is called ‘terahertz (THz)’ radiation. The energy of terahertz radiation corresponds with weak hydrogen or macro–molecular vibration, and the energy band falls in the chemical binding energy of various biomolecules ^14^. In previous research, we found the resonance peak which was associated with methylated DNA ^15^. Because the resonance peak offers information on the characteristic energy between methyl groups and DNA (not covalent bonding energy between the carbons), the radiation energy at the resonance frequency is resonantly absorbed by the molecular vibrations and it may lead to breaking the molecular connection. In the case of methylated DNA, the molecular breakage represents the demethylation. High-power terahertz radiation was used for the breakage whose spectrum was limited around the resonance frequency. The resonant terahertz radiation was irradiated to the isolated DNA of several blood cancers, and the degree of global DNA methylation was decreased without DNA breakage.


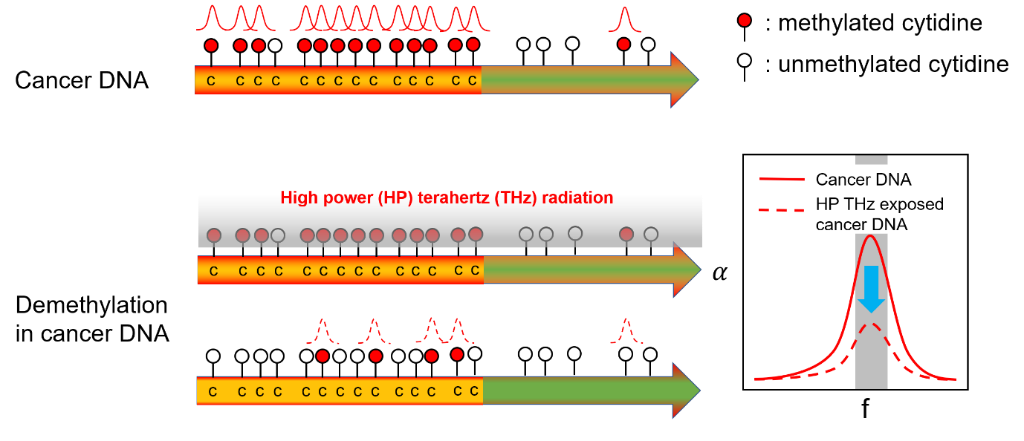


Supplementary Figure 2 The scheme of THz demethylation in cancer DNA. THz radiation transfers the resonant energy into the methylated DNA and the resonant absorption breaks the bonding between methyl groups and DNA sequence. THz demethylation in cancer DNA can work without any chemical process.

**The change of gene expression in living melanoma cells after THz demethylation**

We investigated the chronical change of up-and down-regulation of genes after THz demethylation. Because the modification of apoptosis pathways in tumor cells is a key mechanism of cancer therapy, we checked cancer and apoptosis pathway from RNA of 44629 certain genes in living melanoma cells. We collected each RNA sample after 0 hours (‘immed’ sample), 4 hours, 24 hours, and 48 hours of THz demethylation. We comprehensively evaluated all cancer and apoptosis genes identified in the Kyoto Encyclopedia of Genes and Genomes (KEGG) pathway. The conducted gene set enrichment analysis (GSEA) was performed using KEGG pathways to investigate enriched genes in specific pathways. In GSEA, genes affected by THz demethylation were 98 pathways, including 13 cancer pathways, which were potentially enriched (Suppl. Tab. 1). Significantly different genes between control and THz samples were identified with the cutoff 2-fold change (FC) as the meaningful differentially expressed genes (DEGs) and p-value under 0.0003 of Welch’s T-test. The results of Supplementary Fig 3 suggested that there are several genes involved to induce the modification of cancer and apoptosis pathway. In apoptosis pathway, there was no meaningful change of genes immediately after THz stimulation, but six kinds of genes which included FOS and JUN were up-regulated after 4 hours. After 24 and 48 hours, eleven kinds of genes showed downregulation up to 7-FC. Especially, the expression of FOS and JUN genes family changed dramatically. The cancer pathway acted in a similar trend to apoptosis pathway. The JUN and FOS genes family and CXCL8 gene were modified to down-regulation significantly (more than 4-FC) after 24 hours and 48 hours in cancer pathways though the expression in several genes of the pathways was changed (Suppl.Fig. 3).

**Supplementary Table 1.** Enrichment analysis (GSEA) using KEGG pathways

|  | **GS** | **SIZE** | **ES** | **NES** | **NOM p-val** | **FDR q-val** | **FWER p-val** | **RANK AT MAX** | **LEADING EDGE** |
| --- | --- | --- | --- | --- | --- | --- | --- | --- | --- |
|  | **follow link to MSigDB** |  |  |  |  |  |  |  |  |
| 1 | [KEGG_UBIQUITIN_MEDIATED_PROTEOLYSIS](http://www.gsea-msigdb.org/gsea/msigdb/cards/KEGG_UBIQUITIN_MEDIATED_PROTEOLYSIS) | 135 | 0.28 | 3.3 | 0 | 0 | 0 | 11636 | tags=70%, list=45%, signal=128% |
| 2 | [KEGG_SPLICEOSOME](http://www.gsea-msigdb.org/gsea/msigdb/cards/KEGG_SPLICEOSOME) | 125 | 0.29 | 3.23 | 0 | 0 | 0 | 14059 | tags=83%, list=55%, signal=182% |
| 3 | [KEGG_CELL_CYCLE](http://www.gsea-msigdb.org/gsea/msigdb/cards/KEGG_CELL_CYCLE) | 124 | 0.26 | 2.97 | 0 | 0 | 0 | 11699 | tags=70%, list=45%, signal=128% |
| 4 | [KEGG_RNA_DEGRADATION](http://www.gsea-msigdb.org/gsea/msigdb/cards/KEGG_RNA_DEGRADATION) | 57 | 0.34 | 2.87 | 0 | 0 | 0 | 10125 | tags=70%, list=39%, signal=115% |
| 5 | [KEGG_NUCLEOTIDE_EXCISION_REPAIR](http://www.gsea-msigdb.org/gsea/msigdb/cards/KEGG_NUCLEOTIDE_EXCISION_REPAIR) | 44 | 0.4 | 2.85 | 0 | 0 | 0 | 10383 | tags=80%, list=40%, signal=133% |
| 6 | [KEGG_ADHERENS_JUNCTION](http://www.gsea-msigdb.org/gsea/msigdb/cards/KEGG_ADHERENS_JUNCTION) | 73 | 0.25 | 2.3 | 0 | 0.008 | 0.037 | 13715 | tags=79%, list=53%, signal=169% |
| 7 | [KEGG_RIBOSOME](http://www.gsea-msigdb.org/gsea/msigdb/cards/KEGG_RIBOSOME) | 86 | 0.23 | 2.28 | 0 | 0.007 | 0.04 | 15279 | tags=84%, list=59%, signal=205% |
| 8 | [KEGG_DNA_REPLICATION](http://www.gsea-msigdb.org/gsea/msigdb/cards/KEGG_DNA_REPLICATION) | 36 | 0.33 | 2.2 | 0 | 0.014 | 0.079 | 11637 | tags=78%, list=45%, signal=142% |
| 9 | [KEGG_CHRONIC_MYELOID_LEUKEMIA](http://www.gsea-msigdb.org/gsea/msigdb/cards/KEGG_CHRONIC_MYELOID_LEUKEMIA) | 73 | 0.24 | 2.19 | 0 | 0.012 | 0.08 | 11518 | tags=68%, list=45%, signal=123% |
| 10 | [KEGG_AMINOACYL_TRNA_BIOSYNTHESIS](http://www.gsea-msigdb.org/gsea/msigdb/cards/KEGG_AMINOACYL_TRNA_BIOSYNTHESIS) | 40 | 0.31 | 2.15 | 0.002 | 0.016 | 0.11 | 14529 | tags=88%, list=56%, signal=200% |
| 11 | [KEGG_BASAL_TRANSCRIPTION_FACTORS](http://www.gsea-msigdb.org/gsea/msigdb/cards/KEGG_BASAL_TRANSCRIPTION_FACTORS) | 33 | 0.33 | 2.14 | 0.005 | 0.015 | 0.115 | 10466 | tags=73%, list=41%, signal=122% |
| 12 | [KEGG_OOCYTE_MEIOSIS](http://www.gsea-msigdb.org/gsea/msigdb/cards/KEGG_OOCYTE_MEIOSIS) | 113 | 0.2 | 2.14 | 0 | 0.014 | 0.118 | 10311 | tags=58%, list=40%, signal=97% |
| 13 | [KEGG_PANCREATIC_CANCER](http://www.gsea-msigdb.org/gsea/msigdb/cards/KEGG_PANCREATIC_CANCER) | 69 | 0.23 | 2.03 | 0 | 0.026 | 0.228 | 11746 | tags=67%, list=46%, signal=122% |
| 14 | [KEGG_ENDOMETRIAL_CANCER](http://www.gsea-msigdb.org/gsea/msigdb/cards/KEGG_ENDOMETRIAL_CANCER) | 52 | 0.25 | 1.96 | 0.002 | 0.037 | 0.336 | 11512 | tags=69%, list=45%, signal=125% |
| 15 | [KEGG_HOMOLOGOUS_RECOMBINATION](http://www.gsea-msigdb.org/gsea/msigdb/cards/KEGG_HOMOLOGOUS_RECOMBINATION) | 28 | 0.33 | 1.96 | 0.006 | 0.035 | 0.337 | 10947 | tags=75%, list=42%, signal=130% |
| 16 | [KEGG_SNARE_INTERACTIONS_IN_VESICULAR_TRANSPORT](http://www.gsea-msigdb.org/gsea/msigdb/cards/KEGG_SNARE_INTERACTIONS_IN_VESICULAR_TRANSPORT) | 38 | 0.28 | 1.95 | 0.009 | 0.036 | 0.367 | 14583 | tags=87%, list=57%, signal=200% |
| 17 | [KEGG_OXIDATIVE_PHOSPHORYLATION](http://www.gsea-msigdb.org/gsea/msigdb/cards/KEGG_OXIDATIVE_PHOSPHORYLATION) | 124 | 0.17 | 1.9 | 0.005 | 0.047 | 0.457 | 18990 | tags=93%, list=74%, signal=351% |
| 18 | [KEGG_RENAL_CELL_CARCINOMA](http://www.gsea-msigdb.org/gsea/msigdb/cards/KEGG_RENAL_CELL_CARCINOMA) | 68 | 0.22 | 1.89 | 0.007 | 0.046 | 0.469 | 11746 | tags=68%, list=46%, signal=124% |
| 19 | [KEGG_PARKINSONS_DISEASE](http://www.gsea-msigdb.org/gsea/msigdb/cards/KEGG_PARKINSONS_DISEASE) | 120 | 0.17 | 1.84 | 0.005 | 0.058 | 0.576 | 18779 | tags=92%, list=73%, signal=336% |
| 20 | [KEGG_EPITHELIAL_CELL_SIGNALING_IN_HELICOBACTER_PYLORI_INFECTION](http://www.gsea-msigdb.org/gsea/msigdb/cards/KEGG_EPITHELIAL_CELL_SIGNALING_IN_HELICOBACTER_PYLORI_INFECTION) | 68 | 0.21 | 1.79 | 0.015 | 0.069 | 0.65 | 17074 | tags=88%, list=66%, signal=261% |
| 21 | [KEGG_COLORECTAL_CANCER](http://www.gsea-msigdb.org/gsea/msigdb/cards/KEGG_COLORECTAL_CANCER) | 62 | 0.21 | 1.78 | 0.013 | 0.072 | 0.682 | 11583 | tags=63%, list=45%, signal=114% |
| 22 | [KEGG_LYSINE_DEGRADATION](http://www.gsea-msigdb.org/gsea/msigdb/cards/KEGG_LYSINE_DEGRADATION) | 44 | 0.23 | 1.73 | 0.036 | 0.088 | 0.768 | 11135 | tags=66%, list=43%, signal=116% |
| 23 | [KEGG_SMALL_CELL_LUNG_CANCER](http://www.gsea-msigdb.org/gsea/msigdb/cards/KEGG_SMALL_CELL_LUNG_CANCER) | 84 | 0.18 | 1.73 | 0.018 | 0.086 | 0.774 | 10006 | tags=56%, list=39%, signal=91% |
| 24 | [KEGG_MISMATCH_REPAIR](http://www.gsea-msigdb.org/gsea/msigdb/cards/KEGG_MISMATCH_REPAIR) | 23 | 0.31 | 1.72 | 0.029 | 0.087 | 0.791 | 11335 | tags=70%, list=44%, signal=124% |
| 25 | [KEGG_PYRIMIDINE_METABOLISM](http://www.gsea-msigdb.org/gsea/msigdb/cards/KEGG_PYRIMIDINE_METABOLISM) | 95 | 0.17 | 1.69 | 0.029 | 0.098 | 0.844 | 11685 | tags=62%, list=45%, signal=113% |
| 26 | [KEGG_PORPHYRIN_AND_CHLOROPHYLL_METABOLISM](http://www.gsea-msigdb.org/gsea/msigdb/cards/KEGG_PORPHYRIN_AND_CHLOROPHYLL_METABOLISM) | 32 | 0.27 | 1.68 | 0.032 | 0.097 | 0.852 | 13518 | tags=81%, list=52%, signal=171% |
| 27 | [KEGG_PROTEIN_EXPORT](http://www.gsea-msigdb.org/gsea/msigdb/cards/KEGG_PROTEIN_EXPORT) | 23 | 0.3 | 1.64 | 0.034 | 0.119 | 0.907 | 18115 | tags=100%, list=70%, signal=336% |
| 28 | [KEGG_SELENOAMINO_ACID_METABOLISM](http://www.gsea-msigdb.org/gsea/msigdb/cards/KEGG_SELENOAMINO_ACID_METABOLISM) | 25 | 0.29 | 1.63 | 0.045 | 0.117 | 0.91 | 13090 | tags=80%, list=51%, signal=162% |
| 29 | [KEGG_INSULIN_SIGNALING_PATHWAY](http://www.gsea-msigdb.org/gsea/msigdb/cards/KEGG_INSULIN_SIGNALING_PATHWAY) | 137 | 0.14 | 1.61 | 0.031 | 0.125 | 0.936 | 11461 | tags=57%, list=44%, signal=102% |
| 30 | [KEGG_HUNTINGTONS_DISEASE](http://www.gsea-msigdb.org/gsea/msigdb/cards/KEGG_HUNTINGTONS_DISEASE) | 173 | 0.12 | 1.61 | 0.016 | 0.121 | 0.936 | 14030 | tags=69%, list=54%, signal=150% |
| 31 | [KEGG_PROTEASOME](http://www.gsea-msigdb.org/gsea/msigdb/cards/KEGG_PROTEASOME) | 44 | 0.22 | 1.61 | 0.05 | 0.12 | 0.938 | 16270 | tags=86%, list=63%, signal=234% |
| 32 | [KEGG_RNA_POLYMERASE](http://www.gsea-msigdb.org/gsea/msigdb/cards/KEGG_RNA_POLYMERASE) | 28 | 0.27 | 1.58 | 0.049 | 0.131 | 0.956 | 11372 | tags=71%, list=44%, signal=128% |
| 33 | [KEGG_NEUROTROPHIN_SIGNALING_PATHWAY](http://www.gsea-msigdb.org/gsea/msigdb/cards/KEGG_NEUROTROPHIN_SIGNALING_PATHWAY) | 126 | 0.14 | 1.55 | 0.04 | 0.15 | 0.975 | 11746 | tags=58%, list=46%, signal=106% |
| 34 | [KEGG_N_GLYCAN_BIOSYNTHESIS](http://www.gsea-msigdb.org/gsea/msigdb/cards/KEGG_N_GLYCAN_BIOSYNTHESIS) | 46 | 0.21 | 1.55 | 0.062 | 0.15 | 0.979 | 19803 | tags=98%, list=77%, signal=422% |
| 35 | [KEGG_PROSTATE_CANCER](http://www.gsea-msigdb.org/gsea/msigdb/cards/KEGG_PROSTATE_CANCER) | 89 | 0.16 | 1.53 | 0.05 | 0.154 | 0.981 | 11707 | tags=62%, list=45%, signal=113% |
| 36 | [KEGG_MAPK_SIGNALING_PATHWAY](http://www.gsea-msigdb.org/gsea/msigdb/cards/KEGG_MAPK_SIGNALING_PATHWAY) | 264 | 0.11 | 1.52 | 0.04 | 0.161 | 0.986 | 8689 | tags=43%, list=34%, signal=64% |
| 37 | [KEGG_TASTE_TRANSDUCTION](http://www.gsea-msigdb.org/gsea/msigdb/cards/KEGG_TASTE_TRANSDUCTION) | 51 | 0.18 | 1.49 | 0.053 | 0.177 | 0.995 | 2394 | tags=22%, list=9%, signal=24% |
| 38 | [KEGG_BLADDER_CANCER](http://www.gsea-msigdb.org/gsea/msigdb/cards/KEGG_BLADDER_CANCER) | 41 | 0.2 | 1.43 | 0.079 | 0.225 | 0.999 | 11512 | tags=63%, list=45%, signal=114% |
| 39 | [KEGG_ERBB_SIGNALING_PATHWAY](http://www.gsea-msigdb.org/gsea/msigdb/cards/KEGG_ERBB_SIGNALING_PATHWAY) | 87 | 0.15 | 1.43 | 0.087 | 0.22 | 0.999 | 11512 | tags=59%, list=45%, signal=106% |
| 40 | [KEGG_PROGESTERONE_MEDIATED_OOCYTE_MATURATION](http://www.gsea-msigdb.org/gsea/msigdb/cards/KEGG_PROGESTERONE_MEDIATED_OOCYTE_MATURATION) | 85 | 0.15 | 1.43 | 0.081 | 0.219 | 0.999 | 11737 | tags=60%, list=46%, signal=110% |
| 41 | [KEGG_ENDOCYTOSIS](http://www.gsea-msigdb.org/gsea/msigdb/cards/KEGG_ENDOCYTOSIS) | 180 | 0.11 | 1.43 | 0.057 | 0.214 | 0.999 | 7801 | tags=39%, list=30%, signal=55% |
| 42 | [KEGG_VIBRIO_CHOLERAE_INFECTION](http://www.gsea-msigdb.org/gsea/msigdb/cards/KEGG_VIBRIO_CHOLERAE_INFECTION) | 54 | 0.18 | 1.42 | 0.094 | 0.222 | 1 | 16327 | tags=83%, list=63%, signal=227% |
| 43 | [KEGG_THYROID_CANCER](http://www.gsea-msigdb.org/gsea/msigdb/cards/KEGG_THYROID_CANCER) | 29 | 0.22 | 1.37 | 0.121 | 0.268 | 1 | 11429 | tags=66%, list=44%, signal=118% |
| 44 | [KEGG_NON_SMALL_CELL_LUNG_CANCER](http://www.gsea-msigdb.org/gsea/msigdb/cards/KEGG_NON_SMALL_CELL_LUNG_CANCER) | 54 | 0.17 | 1.37 | 0.138 | 0.263 | 1 | 10030 | tags=54%, list=39%, signal=88% |
| 45 | [KEGG_BASE_EXCISION_REPAIR](http://www.gsea-msigdb.org/gsea/msigdb/cards/KEGG_BASE_EXCISION_REPAIR) | 33 | 0.21 | 1.34 | 0.153 | 0.283 | 1 | 16044 | tags=85%, list=62%, signal=225% |
| 46 | [KEGG_PENTOSE_AND_GLUCURONATE_INTERCONVERSIONS](http://www.gsea-msigdb.org/gsea/msigdb/cards/KEGG_PENTOSE_AND_GLUCURONATE_INTERCONVERSIONS) | 19 | 0.27 | 1.34 | 0.148 | 0.282 | 1 | 13228 | tags=79%, list=51%, signal=162% |
| 47 | [KEGG_INOSITOL_PHOSPHATE_METABOLISM](http://www.gsea-msigdb.org/gsea/msigdb/cards/KEGG_INOSITOL_PHOSPHATE_METABOLISM) | 54 | 0.17 | 1.34 | 0.131 | 0.279 | 1 | 7242 | tags=41%, list=28%, signal=57% |
| 48 | [KEGG_MTOR_SIGNALING_PATHWAY](http://www.gsea-msigdb.org/gsea/msigdb/cards/KEGG_MTOR_SIGNALING_PATHWAY) | 51 | 0.17 | 1.32 | 0.133 | 0.294 | 1 | 11550 | tags=63%, list=45%, signal=113% |
| 49 | [KEGG_PATHOGENIC_ESCHERICHIA_COLI_INFECTION](http://www.gsea-msigdb.org/gsea/msigdb/cards/KEGG_PATHOGENIC_ESCHERICHIA_COLI_INFECTION) | 55 | 0.16 | 1.29 | 0.135 | 0.328 | 1 | 15022 | tags=76%, list=58%, signal=183% |
| 50 | [KEGG_GLIOMA](http://www.gsea-msigdb.org/gsea/msigdb/cards/KEGG_GLIOMA) | 65 | 0.15 | 1.29 | 0.15 | 0.322 | 1 | 11007 | tags=57%, list=43%, signal=99% |
| 51 | KEGG_PURINE_METABOLISM | 156 | 0.11 | 1.28 | 0.136 | 0.322 | 1 | 10130 | tags=49%, list=39%, signal=80% |
| 52 | KEGG_FRUCTOSE_AND_MANNOSE_METABOLISM | 33 | 0.2 | 1.27 | 0.166 | 0.324 | 1 | 17295 | tags=88%, list=67%, signal=267% |
| 53 | KEGG_T_CELL_RECEPTOR_SIGNALING_PATHWAY | 108 | 0.12 | 1.27 | 0.154 | 0.325 | 1 | 7904 | tags=41%, list=31%, signal=59% |
| 54 | KEGG_ONE_CARBON_POOL_BY_FOLATE | 16 | 0.27 | 1.26 | 0.178 | 0.324 | 1 | 15336 | tags=88%, list=60%, signal=216% |
| 55 | KEGG_CYSTEINE_AND_METHIONINE_METABOLISM | 33 | 0.19 | 1.25 | 0.149 | 0.33 | 1 | 16475 | tags=85%, list=64%, signal=235% |
| 56 | KEGG_CITRATE_CYCLE_TCA_CYCLE | 30 | 0.2 | 1.24 | 0.2 | 0.34 | 1 | 16598 | tags=87%, list=64%, signal=243% |
| 57 | KEGG_REGULATION_OF_AUTOPHAGY | 34 | 0.19 | 1.24 | 0.201 | 0.339 | 1 | 11161 | tags=65%, list=43%, signal=114% |
| 58 | KEGG_FC_GAMMA_R_MEDIATED_PHAGOCYTOSIS | 94 | 0.12 | 1.23 | 0.194 | 0.344 | 1 | 10006 | tags=49%, list=39%, signal=80% |
| 59 | KEGG_AMINO_SUGAR_AND_NUCLEOTIDE_SUGAR_METABOLISM | 43 | 0.16 | 1.23 | 0.211 | 0.341 | 1 | 17295 | tags=86%, list=67%, signal=261% |
| 60 | KEGG_APOPTOSIS | 87 | 0.12 | 1.22 | 0.205 | 0.342 | 1 | 11000 | tags=54%, list=43%, signal=94% |
| 61 | KEGG_NICOTINATE_AND_NICOTINAMIDE_METABOLISM | 24 | 0.22 | 1.21 | 0.223 | 0.348 | 1 | 8770 | tags=54%, list=34%, signal=82% |
| 62 | KEGG_B_CELL_RECEPTOR_SIGNALING_PATHWAY | 75 | 0.13 | 1.2 | 0.242 | 0.361 | 1 | 10030 | tags=52%, list=39%, signal=85% |
| 63 | KEGG_ALZHEIMERS_DISEASE | 159 | 0.1 | 1.2 | 0.222 | 0.357 | 1 | 21535 | tags=95%, list=84%, signal=574% |
| 64 | KEGG_ACUTE_MYELOID_LEUKEMIA | 57 | 0.14 | 1.2 | 0.248 | 0.352 | 1 | 10425 | tags=54%, list=40%, signal=91% |
| 65 | KEGG_PHOSPHATIDYLINOSITOL_SIGNALING_SYSTEM | 76 | 0.13 | 1.16 | 0.272 | 0.398 | 1 | 8510 | tags=43%, list=33%, signal=65% |
| 66 | KEGG_BIOSYNTHESIS_OF_UNSATURATED_FATTY_ACIDS | 22 | 0.22 | 1.15 | 0.266 | 0.405 | 1 | 20160 | tags=100%, list=78%, signal=459% |
| 67 | KEGG_WNT_SIGNALING_PATHWAY | 151 | 0.09 | 1.13 | 0.278 | 0.43 | 1 | 8919 | tags=42%, list=35%, signal=64% |
| 68 | KEGG_NOD_LIKE_RECEPTOR_SIGNALING_PATHWAY | 62 | 0.13 | 1.13 | 0.306 | 0.428 | 1 | 8822 | tags=44%, list=34%, signal=66% |
| 69 | KEGG_FATTY_ACID_METABOLISM | 42 | 0.16 | 1.12 | 0.309 | 0.438 | 1 | 15845 | tags=81%, list=61%, signal=210% |
| 70 | KEGG_VALINE_LEUCINE_AND_ISOLEUCINE_DEGRADATION | 43 | 0.15 | 1.07 | 0.365 | 0.506 | 1 | 11469 | tags=58%, list=45%, signal=105% |
| 71 | KEGG_PATHWAYS_IN_CANCER | 322 | 0.07 | 1.07 | 0.366 | 0.501 | 1 | 11756 | tags=52%, list=46%, signal=95% |
| 72 | KEGG_PROPANOATE_METABOLISM | 32 | 0.17 | 1.07 | 0.376 | 0.495 | 1 | 18454 | tags=91%, list=72%, signal=319% |
| 73 | KEGG_RIG_I_LIKE_RECEPTOR_SIGNALING_PATHWAY | 71 | 0.11 | 1.04 | 0.411 | 0.536 | 1 | 7573 | tags=39%, list=29%, signal=56% |
| 74 | KEGG_GNRH_SIGNALING_PATHWAY | 99 | 0.1 | 1.03 | 0.411 | 0.531 | 1 | 10311 | tags=49%, list=40%, signal=82% |
| 75 | KEGG_GLYOXYLATE_AND_DICARBOXYLATE_METABOLISM | 16 | 0.23 | 1.03 | 0.383 | 0.529 | 1 | 17903 | tags=94%, list=69%, signal=307% |
| 76 | KEGG_FC_EPSILON_RI_SIGNALING_PATHWAY | 77 | 0.11 | 0.99 | 0.443 | 0.582 | 1 | 10030 | tags=49%, list=39%, signal=81% |
| 77 | KEGG_LONG_TERM_POTENTIATION | 70 | 0.11 | 0.99 | 0.476 | 0.584 | 1 | 8154 | tags=41%, list=32%, signal=60% |
| 78 | KEGG_TOLL_LIKE_RECEPTOR_SIGNALING_PATHWAY | 101 | 0.09 | 0.98 | 0.479 | 0.589 | 1 | 17216 | tags=80%, list=67%, signal=241% |
| 79 | KEGG_AMYOTROPHIC_LATERAL_SCLEROSIS_ALS | 53 | 0.12 | 0.96 | 0.45 | 0.617 | 1 | 19680 | tags=91%, list=76%, signal=382% |
| 80 | KEGG_GLYCOSYLPHOSPHATIDYLINOSITOL_GPI_ANCHOR_BIOSYNTHESIS | 25 | 0.16 | 0.95 | 0.511 | 0.635 | 1 | 18041 | tags=88%, list=70%, signal=293% |
| 81 | KEGG_VASOPRESSIN_REGULATED_WATER_REABSORPTION | 44 | 0.13 | 0.93 | 0.542 | 0.661 | 1 | 15330 | tags=75%, list=59%, signal=185% |
| 82 | KEGG_NOTCH_SIGNALING_PATHWAY | 47 | 0.12 | 0.92 | 0.556 | 0.665 | 1 | 17498 | tags=83%, list=68%, signal=258% |
| 83 | KEGG_STARCH_AND_SUCROSE_METABOLISM | 41 | 0.13 | 0.89 | 0.572 | 0.704 | 1 | 20993 | tags=95%, list=81%, signal=512% |
| 84 | KEGG_LONG_TERM_DEPRESSION | 68 | 0.1 | 0.86 | 0.625 | 0.743 | 1 | 10311 | tags=50%, list=40%, signal=83% |
| 85 | KEGG_P53_SIGNALING_PATHWAY | 68 | 0.1 | 0.85 | 0.67 | 0.747 | 1 | 11449 | tags=54%, list=44%, signal=98% |
| 86 | KEGG_RIBOFLAVIN_METABOLISM | 16 | 0.19 | 0.85 | 0.657 | 0.745 | 1 | 20993 | tags=100%, list=81%, signal=539% |
| 87 | KEGG_DRUG_METABOLISM_OTHER_ENZYMES | 42 | 0.12 | 0.85 | 0.653 | 0.741 | 1 | 7716 | tags=40%, list=30%, signal=58% |
| 88 | KEGG_PRIMARY_BILE_ACID_BIOSYNTHESIS | 16 | 0.18 | 0.84 | 0.634 | 0.75 | 1 | 17428 | tags=88%, list=68%, signal=270% |
| 89 | KEGG_NITROGEN_METABOLISM | 23 | 0.14 | 0.79 | 0.735 | 0.819 | 1 | 4035 | tags=26%, list=16%, signal=31% |
| 90 | KEGG_GLYCOSAMINOGLYCAN_BIOSYNTHESIS_CHONDROITIN_SULFATE | 22 | 0.14 | 0.77 | 0.75 | 0.829 | 1 | 22111 | tags=100%, list=86%, signal=704% |
| 91 | KEGG_ASCORBATE_AND_ALDARATE_METABOLISM | 16 | 0.16 | 0.73 | 0.787 | 0.875 | 1 | 13228 | tags=69%, list=51%, signal=141% |
| 92 | KEGG_TERPENOID_BACKBONE_BIOSYNTHESIS | 15 | 0.16 | 0.73 | 0.801 | 0.871 | 1 | 14128 | tags=73%, list=55%, signal=162% |
| 93 | KEGG_BETA_ALANINE_METABOLISM | 22 | 0.14 | 0.73 | 0.8 | 0.863 | 1 | 13084 | tags=68%, list=51%, signal=138% |
| 94 | KEGG_TGF_BETA_SIGNALING_PATHWAY | 86 | 0.07 | 0.73 | 0.813 | 0.858 | 1 | 11477 | tags=52%, list=45%, signal=94% |
| 95 | KEGG_ADIPOCYTOKINE_SIGNALING_PATHWAY | 67 | 0.07 | 0.64 | 0.924 | 0.937 | 1 | 23338 | tags=99%, list=91%, signal=1041% |
| 96 | KEGG_CYTOSOLIC_DNA_SENSING_PATHWAY | 55 | 0.07 | 0.59 | 0.952 | 0.969 | 1 | 20559 | tags=91%, list=80%, signal=449% |
| 97 | KEGG_PRION_DISEASES | 35 | 0.08 | 0.53 | 0.957 | 0.988 | 1 | 10780 | tags=51%, list=42%, signal=88% |
| 98 | KEGG_VEGF_SIGNALING_PATHWAY | 74 | 0.06 | 0.51 | 0.98 | 0.985 | 1 | 19976 | tags=86%, list=78%, signal=384% |


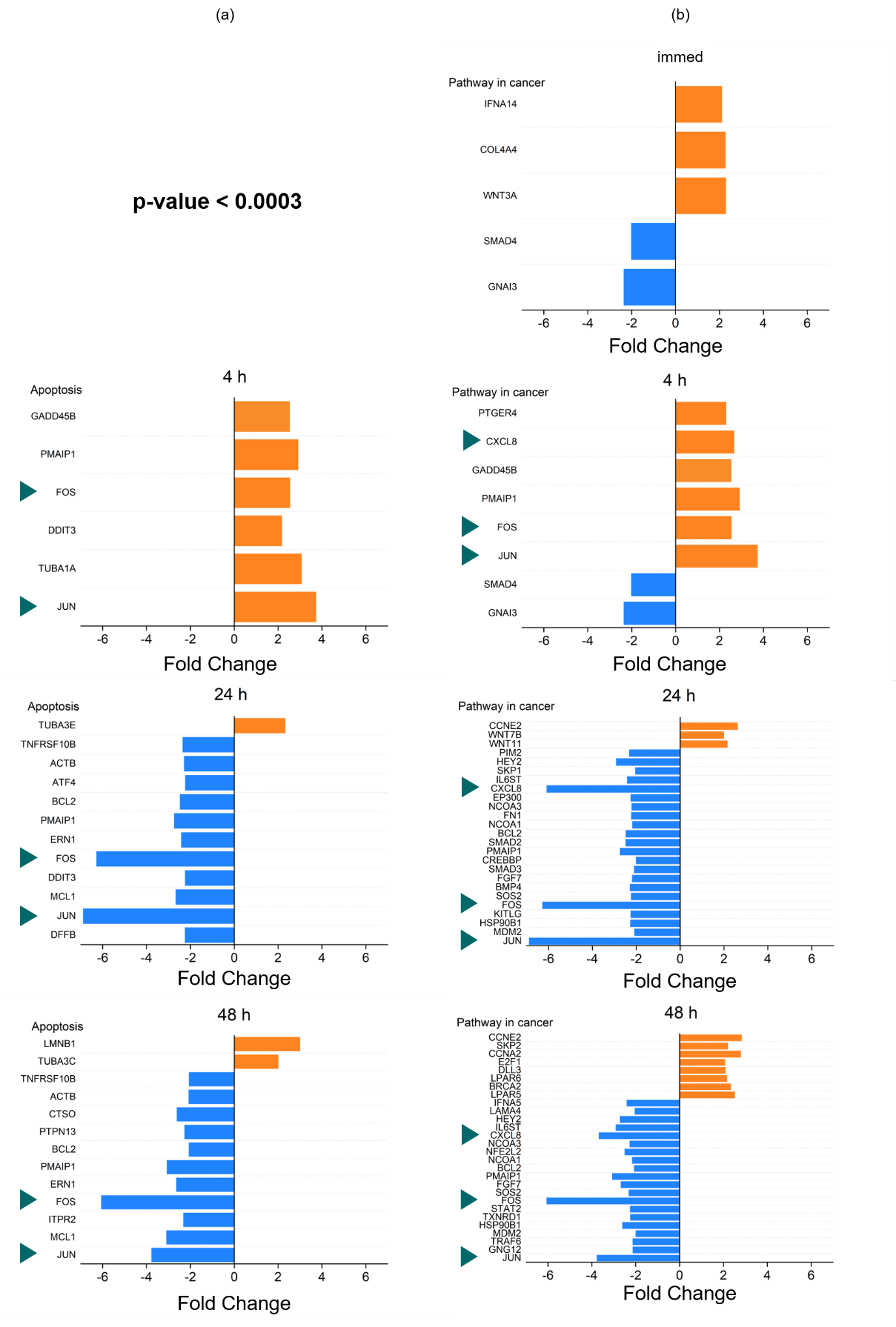


Supplementary Figure 3. Relative expression in identified genes of (a) apoptosis and (b) cancer (pathway in cancer) pathway (p-value < 0.0003). THz demethylation occurred the modification of gene expression within the living cell.

**Passive and active demethylation**

Passive demethylation is dependent on genomic heredity. The molecules in these drugs are inserted into DNA strands as a replication of cytidine. DNMTs are bound to the molecules rather than cytidine when DNMTs approach DNA strands to copy the methylation condition to the daughter strands. These processes are repeated during cell division and the degree of DNA methylation is diluted along the processes. The passive demethylation drugs such as Vidaza^TM^ (5-Azacytidine) or Decitabine^TM^ (2’-dexoy-5-azacytidine) inhibit DNMT activity and allow the re-expression of tumor suppressors and this is known as the mechanism of passive demethylation (Suppl. Fig. 4). The approval for passive demethylation agents has given hope to the researchers studying epigenetic therapeutic applications, but the problem of side effects will still remain and it always requires the replicant process. However, the active demethylation technique, which is the replication-independent method, might regulate the DNA methylation delicately because it removes methyl groups from DNA directly and it may be a solution to achieve both prevention of cancer growth and unleashing tumor suppressors with fewer side effects. The ten–eleven translocation (TET) family of methylated cytosine dioxygenases consisting of TET1, TET2, and TET3 is widely used in active demethylation. The TET enzymes catalyze the hydroxylation of 5-mC to 5-hydroxymethylcytosine (5-hmC), and oxidize 5-hmC to generate 5-formylcytosine (5-fC) and 5-carboxycytosine (5-caC) ^16-18^. The oxidized cytosines (5-fC and 5-caC) are recognized as apurinic/apyrimidinic (AP) sites and then removed through the DNA repair pathway of thymine DNA glycosylase (TDG) and replaced by cytosine in the DNA base sequence. Finally, the activity of TET enzymes exchanges 5-mC to nonmethylated cytosine, and it is the known mechanism of active methylation (Suppl. Fig. 5). Unfortunately, there are no techniques and agents for active demethylation in clinical trials yet. The mechanism of active demethylation is known relatively less than passive demethylation. Several researchers are trying to apply some agents and enzymes in order to achieve active demethylation ^19,20^.

In the case of cancer biology, demethylation modulates cancer development in various pathways including cell growth, apoptosis, and signaling pathways by manipulating gene expression. Thus, THz demethylation may be a key to novel molecular cancer therapy (Suppl. Fig. 6). The principle for THz demethylation is still under study both physically and biologically, but it might develop into a unique non-contact technique to achieve active demethylation.


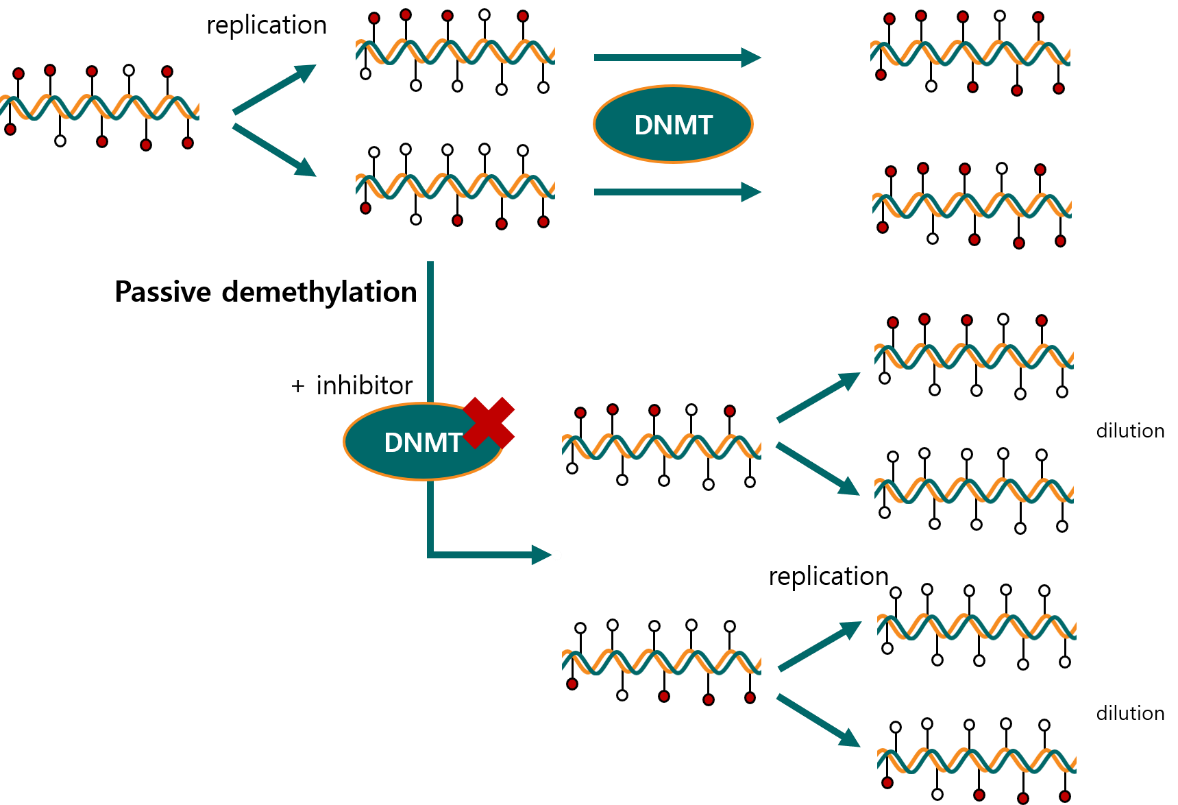


Supplementary Figure 4. Mechanism of passive demethylation. In living organism, original DNA molecules are replicated to hemi-methylated DNA. DNMT enzyme restores hemi-methylated DNA to original methylation. Inhibition of DNMT prevents the restoring process and leads to unmethylated DNA in cell division. Daughter cells have lesser DNA methylation than the mother cells.


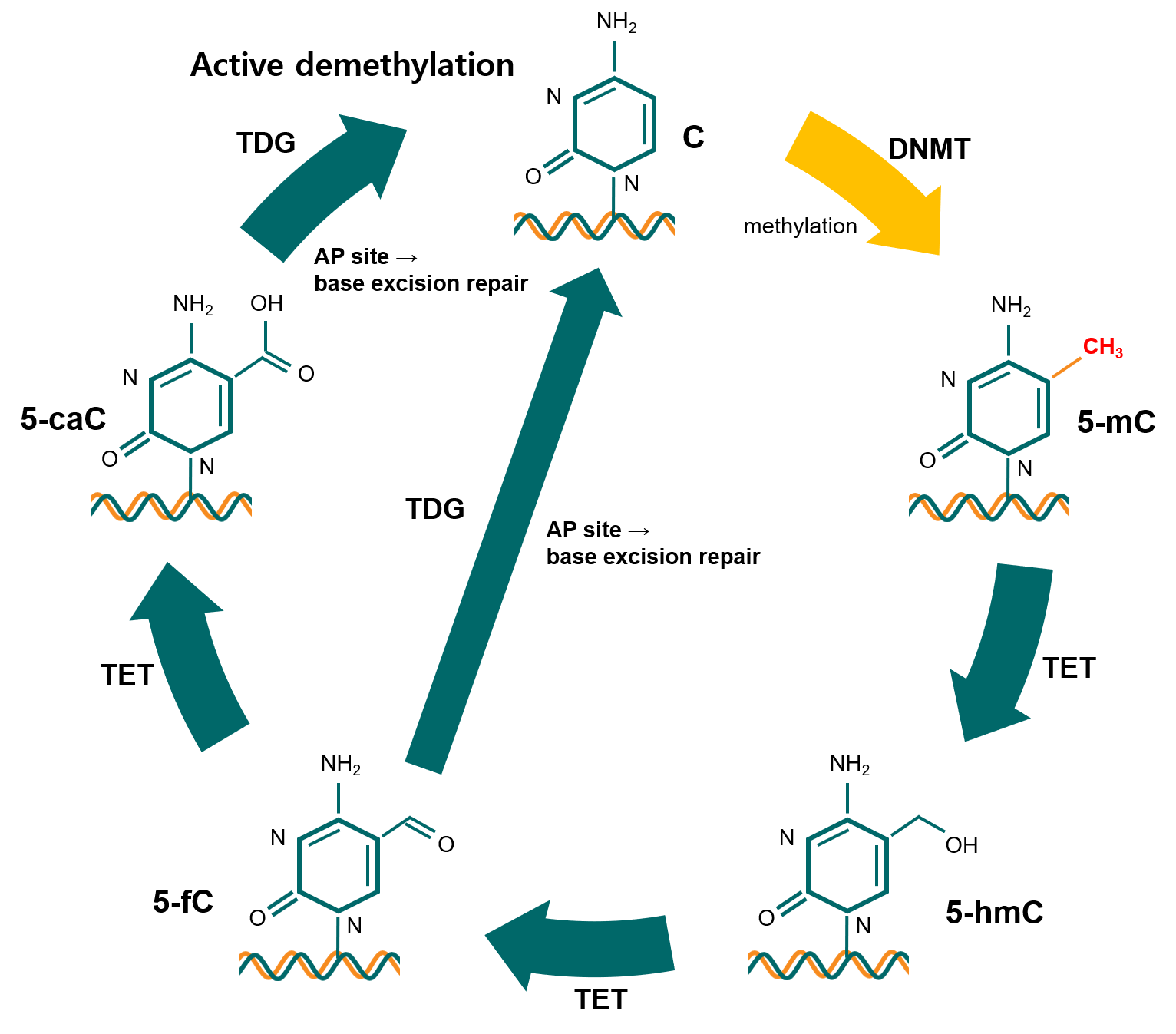


Supplementary Figure 5. Mechanism of active demethylation in chemical and physical processes. TET enzymes convert 5-mC (5-methylcytosine) into 5-hmC, 5-fC, and 5-caC, which are analogs. The oxidation products of 5-mC are removed via base excision repair process using TDG. As a result, 5-mC is removed from DNA sequence without replicant process.


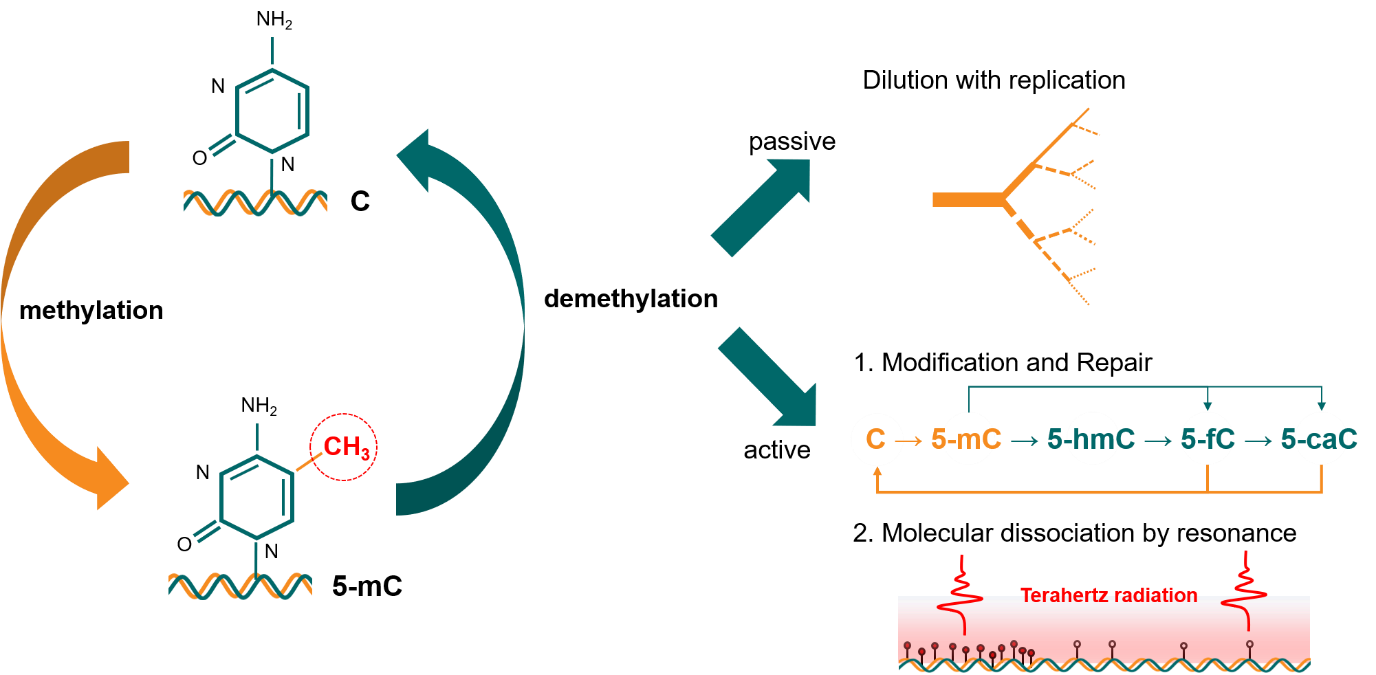


Supplementary Figure 6. Passive and active demethylation to remove methyl groups from cytosine of DNA. Passive demethylation uses an epigenetic delusion with the replicant process of cell division. On the other hand, active demethylation is the replication-independent process. Active demethylation utilizes a chemical process with enzymes or a physical process using terahertz radiation.

**Supplementary References**

1. Bloushtain-Qimron, N., *et al.* Cell type-specific DNA methylation patterns in the human breast. *Proc. Natl. Acad. Sci. U. S. A.* **105**(37), 14076–14081 (2008).

2. Moore, L. D., Le, T., & Fan, G. DNA methylation and its basic function. *Neuropsychopharmacology* **38**(1), 23–38, (2013).

3. Cheishvili, D., Boureau, L., & Szyf, M. DNA demethylation and invasive cancer: Implications for therapeutics. *Br. J. Pharmacol.* **172**(11), 2705–2715 (2015).

4. Portela, A. & Esteller, M. Epigenetic modifications and human disease. *Nat. Biotechnol.* **28**(10), 1057–1068 (2010).

5. Linnekamp, J. F., Butter, R., Spijker, R., Medema, J. P., & van Laarhoven, H. W. M. Clinical and biological effects of demethylating agents on solid tumours – A systematic review. *Cancer Treat. Rev.* **54**, 10–23 (2017).

6. Widschwendter, M., *et al.* Association of breast cancer DNA methylation profiles with hormone receptor status and response to tamoxifen. *Cancer Res.* **64**(11), 3807–3813 (2004).

7. Makos, M., *et al.* Distinct hypermethylation patterns occur at altered chromosome loci in human lung and colon cancer. *Proc. Natl. Acad. Sci. U. S. A.* **89**(5), 1929–1933 (1992).

8. Ehrlich, M. DNA methylation in cancer: too much, but also too little. *Oncogene* **21**(35), 5400–5413 (2002).

9. Kulis, M. & Esteller, M. DNA methylation and cancer. *Adv. Genet.* **70**, 27–56 (2010).

10. Sheaffer, K. L., Elliott, E. N., & Kaestner, K. H. DNA hypomethylation contributes to genomic instability and intestinal cancer initiation. *Cancer Prev. Res. (Phila)* **9**(7), 534–546 (2016).

11. Ehrlich, M. DNA hypomethylation in cancer cells. *Epigenomics* **1**(2), 239–259 (2009).

12. Fabiani, E., *et al.* Analysis of genome-wide methylation and gene expression induced by 5-aza-2′-deoxycytidine identifies BCL2L10 as a frequent methylation target in acute myeloid leukemia. *Leuk. Lymphoma* **51**(12), 2275–2284 (2010).

13. Agrawal, K., Das, V., Vyas, P., & Hajdúch, M. Nucleosidic DNA demethylating epigenetic drugs – A comprehensive review from discovery to clinic. *Pharmacol. Ther.* **188**, 45–79 (2018).

14. Cheon, H., Yang, H.-J., & Son, J.-H. Toward clinical cancer imaging using terahertz spectroscopy. *IEEE J. Sel. Top. Quantum Electron.* **23**, 8600109 (2017).

15. Cheon, H., Yang, H.J., Lee, S.H., Kim, Y.A., & Son, J.H. Terahertz molecular resonance of cancer DNA. *Sci. Rep.* **6**, 37103 (2016).

16. Ooi, S.K.T. & Bestor, T.H. The colorful history of active DNA demethylation. Cell 133, 1145–1148 (2008).

17. Scourzic, L., Mouly, E. & Bernard, O.A. TET proteins and the control of cytosine demethylation in cancer. Genome Med. 7, 1–16 (2015).

18. Cheng, Y., He, C., Wang, M., Ma, X., Mo, F., Yang, S., Han, J. & Wei, X. Targeting epigenetic regulators for cancer therapy: Mechanisms and advances in clinical trials. Signal Transduct. Target. Ther. 4, 1–39 (2019).

19. Chia, N., Wang, L., & Ruden, D.M. TET inhibitors as potential new cancer drugs - An enzyme that Converts 5-methylcytosine to 5-hydroxymethylcytosine. *Int. Drug Discov.* **2012**, 21–23 (2012).

20. Detich, N., Bovenzi, V., & Szyf, M. Valproate induces replication-independent active DNA demethylation. *J. Biol. Chem.* **278**(30), 27586–27592 (2003).
